# Simple practical method for synthesis of trisubstituted imidazoles: an efficient copper catalyzed multicomponent reaction†

**DOI:** 10.1039/d1ra01767e

**Published:** 2021-06-22

**Authors:** Vikas D. Kadu, Ganesh A. Mali, Siddheshwar P. Khadul, Gokul J. Kothe

**Affiliations:** School of Chemical Sciences, Punyashlok Ahilyadevi Holkar Solapur University Solapur-413255 Maharashtra India vikaskadu1@gmail.com

## Abstract

A rapid practical process has been developed for synthesis of 2,4,5-trisubstituted-imidazoles in excellent yields up to 95% from readily available starting materials. In this CuI catalyzed synthesis, trisubstituted imidazoles were afforded in short reaction times, wherein the substrate scope is well explored with benzoin as well as benzil reacting with different aldehydes in the presence of ammonium acetate as the nitrogen source.

## Abstract 2

A rapid practical process has been developed for synthesis of 2,4,5-trisubstituted-imidazoles in excellent yields up to 95% from readily available starting materials.

## Graphical abstract

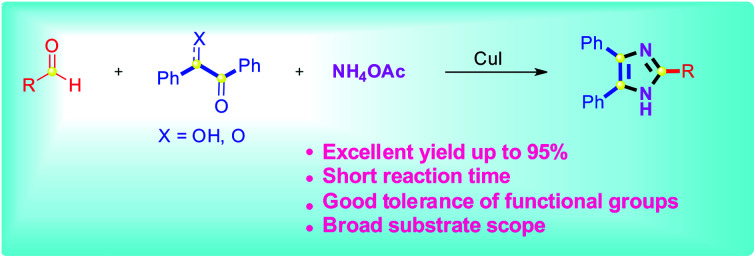

## Introduction

The five-membered aromatic imidazole ring is an important heterocycle broadly present in various natural products and synthetic molecules with a broad range of medicinal applications.^1–7^ Furthermore, they possess diverse biological properties such as antibacterial, antifungal, anticancer, anti-inflammatory, antihistaminic, antitubercular, antihypertensive, antiviral, antineuropathic, anti-obesity, antiparasitic and have possible applications in pathology and diagnostics.^6^ The imidazole core is an important aromatic heterocycle which has broad therapeutic interest due to its two nitrogen atoms leading to hydrogen bond creation for improving water solubility properties. Further, the imidazole nucleus has been recognized as an important isostere of amide, thiazole, tetrazole, pyrazole, oxazole and triazole with an attractive binding site available for interacting with various biomolecules, anions and cations in biological systems offering potential in imidazole-based drug discovery and developments.^8^ Furthermore, imidazole has good photophysical properties,^9–17^ and is used as a ligand in homogeneous catalysis^18–20^ and functionalized materials.^21–30^

Several imidazole-based scaffolds have been widely utilized in the clinic for treatment of different diseases with great therapeutic potency, which have proved the great development significance. Imidazole-based medicinal chemistry research is rapidly becoming an active area, and chemists are encouraged by these imidazole-linked drugs as enormous therapeutic assets. Moreover, it is gaining importance in agrochemicals, solar cell dyes, and functional materials; the process development has good functional group tolerance and substrate flexibility is increasing. Several methods have been reported for synthesis of trisubstituted imidazoles.^31^ But, alternative method development for regiocontrolled substituted imidazoles has strategic importance due to a few shortcomings such as complex starting materials, use of strong oxidants, requirement of stoichiometric amounts of toxic metals, microwave conditions, byproducts formation and tedious work up procedures. The importance of economic viability and nature balance means the development of environmentally benign, simple and fast protocols for synthesis of polysubstituted imidazoles still has significant scope. To continue efforts towards synthesis of nitrogen containing heterocycles,^32–36^ herein we are going to report an alternative simple and efficient method for the synthesis of polysubstituted imidazoles.

## Results and discussion

We started by investigating 4-chlorobenzaldehyde 1a (1 mmol), benzoin 2a (1 mmol) and ammonium acetate 3 (3 mmol) as model substrates in the presence of (20 mol%) Cu(i) catalysts. We screened CuCl, CuBr and CuI catalysts; where 75% yield of the anticipated product 4a was obtained after 90 min in DMSO as reaction medium with CuI catalyst at 140 °C (Table 1, entries 1–3). From these results, the reaction was further optimized by using CuI (20 mol%) with various solvents. Initially, the reaction was carried out in DMF at 140 °C temperature which afforded desired product 4a in 65% yield (Table 1, entry 4). By considering green chemistry principles and keeping in mind their pharmaceutical importance, the reaction conditions were further optimized using green solvents to find the best conditions. Gratifyingly, when it was optimized to use butanol solvent at reflux, the product 4a was afforded in 85% yield within 20 min (Table 1, entry 5). After that, the reaction was investigated with ethanol solvent at reflux; where the anticipated product 4a was obtained in 76% yield in a slightly greater time up to 70 min (Table 1, entry 6). Moreover, the reaction studied with methanol solvent refluxed at 65 °C procured the product 4a in 74% yield after 90 min (Table 1, entry 7).

**Table 1:** Optimization of reaction conditions^a^

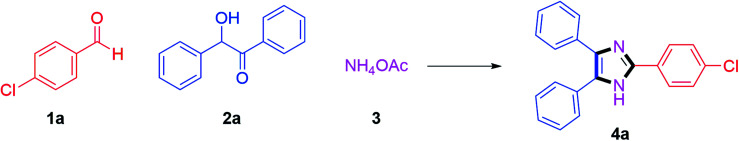					
Entry	Catalyst	Solvent	Temp (°C)	Time (min)	Yield^b^ (%)
1	CuCl	DMSO	140	90 min	60
2	CuBr	DMSO	140	90 min	65
3	CuI	DMSO	140	90 min	75
4	CuI	DMF	140	90 min	65
5	CuI	BuOH	reflux	20 min	85
6	CuI	EtOH	reflux	70 min	76
7	CuI	MeOH	reflux	90 min	74
8	CuI	H_2_O	reflux	90 min	10
9	CuI	Toluene	110	90 min	67
10	CuI	Chlorobenzene	120	90 min	56
11	CuI	CH_3_CN	reflux	90 min	68
12^c^	CuI	BuOH	reflux	20 min	85
13^d^	CuI	BuOH	reflux	25 min	84
14^e^	CuI	BuOH	reflux	30 min	83
15^c^	CuI	BuOH	90	80 min	80
16^c^	CuI	BuOH	70	80 min	78
17^c^	CuI	—	100	90 min	64

a Reaction conditions: 1a (1.0 mmol), 2a (1.0 mmol), 3 (3 mmol), CuI (20 mol%), solvent (7 mL).

b Isolated yield.

c 15 mol% CuI catalyst was used.

d 10 mol% CuI catalyst was used.

e 5 mol% CuI catalyst was used.

In the next step, the reaction was examined with water solvent under refluxed conditions; but 4a product formation was observed only in up to 10% yield with 90 min reaction time (Table 1, entry 8). Then, the reaction conditions were optimized with nonpolar solvents such as toluene (at 110 °C) and chlorobenzene (at 120 °C) which afforded the product 4a in yields of 67% and 56% respectively (Table 1, entries 9–10). Furthermore, the reaction carried out in CH_3_CN solvent at reflux obtained a yield of product 4a of up to 68% in 90 min (Table 1, entry 11). To find out the best optimized conditions, the catalyst loading was examined, and the same results were reproduced with 15 mol% CuI catalyst (Table 1, entry 12). Further, the reaction was also studied in the presence of 10 mol% and 5 mol% CuI catalyst, which resulted in slightly lower yields of the desired product in 25 min and 30 min reaction times respectively (Table 1, entry 13–14). Next, we investigated the temperature effects and the product 4a was obtained in lower yields of 80% and 78% after 80 min for 90 °C and 70 °C respectively (Table 1, entries 15–16). After that, the reaction was studied in neat reaction conditions; where the anticipated product 4a formed in up to 64% yield (Table 1, entry 17). Therefore, the reaction conditions described in entry 12 were noted to be optimal, tolerating for maximum conversion to the desired trisubstituted imidazole product 4a.

By using the optimized reaction conditions, we explored the substrate scope for benzoin 2a against various aryl, heteroaryl and aliphatic aldehydes, and the results are depicted in Table 2. The different functional groups such as alkyl, halogens, nitro, methoxy and hydroxyl were found feasible under the optimized reaction conditions. The aromatic aldehydes furnished the desired products in good to high yields with both electron-donating and electron-withdrawing groups. The *p*-substituted electron-donating and electron-withdrawing aryl aldehydes such as 4-Cl, 4-OMe, 4-OH, 4-CN and 4-NO_2_ were studied and afforded the desired products 4a (85%), 4b (94%), 4c (92%), 4e (84%) and 4f (92%) (Table 2). Furthermore, the sterically hindered, *o*-substituted aryl aldehydes such as 2-F and 2-NO_2_ furnished products 4g (89%) and 4h (95%) (Table 2, entries 7–8). Then, the *m*-substituted (3-OMe and 3-NO_2_) aryl aldehydes were also explored and provided the preferred products (4i and 4j) in 92% and 96% yields respectively (Table 2, entries 9–10). After that, the substrate scope was extended with disubstituted functional groups; where the trisubstituted imidazoles were afforded in excellent yields of 92% (4k; 2-Cl, 4-Cl), 94% (4l; 2-Cl, 6-F), 90% (4m; 3-OMe, 4-OH), 92% (4n; 2,5-OMe) and 94% (4o; 3,4-OMe) (Table 2, entries 11–15). In addition, the optimized conditions were used for 1-naphthaldehyde and heteroaryl aldehydes (1*H*-indole-3-carbaldehyde, picolinaldehyde and thiophene-2-carbaldehyde) which provided the products in significant yields: 4p (95%), 4q (84%), 4r (83%) and 4s (88%) (Table 2, entries 16–19).

**Table 2:** Substrate scope with benzoin for trisubstituted imidazole synthesis^a^

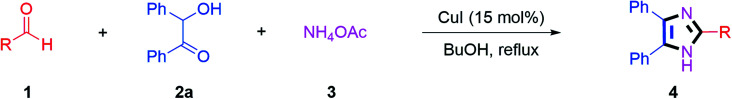							
Entry	4	Time (min)	Yield^b^ (%)	Entry	4	Time (min)	Yield^b^ (%)
1	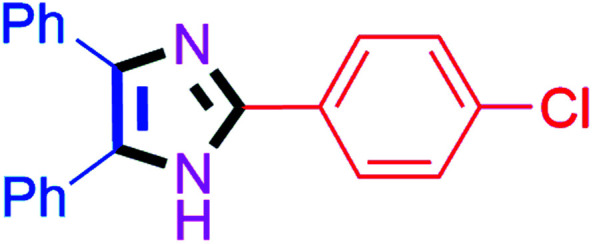	20	4a (85%)	11	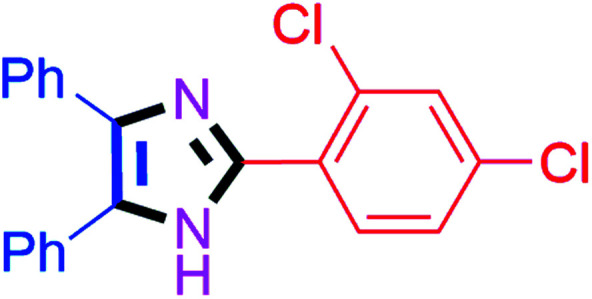	40	4k (92%)
2	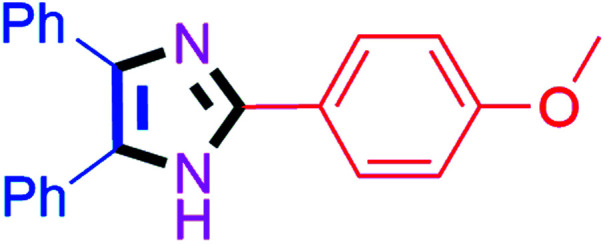	20	4b (94%)	12	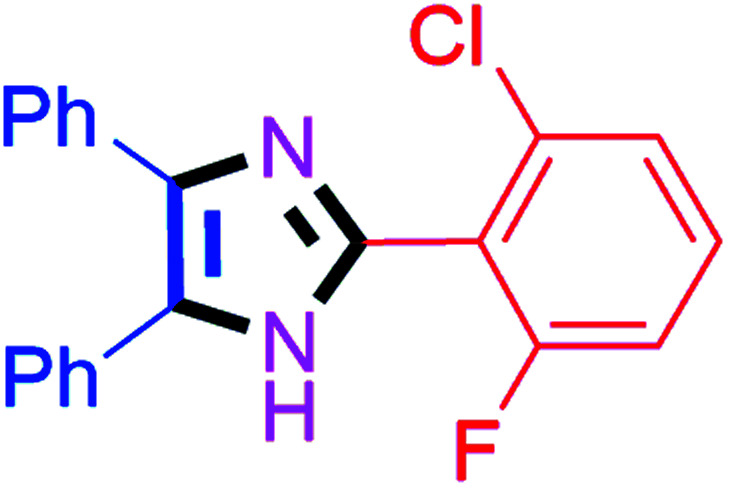	45	4l (94%)
3	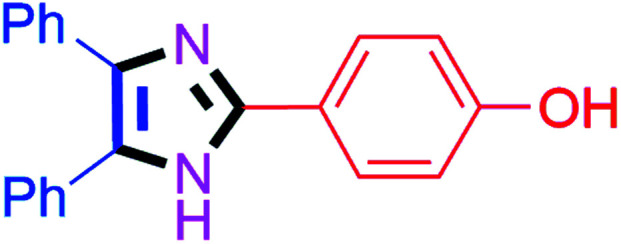	30	4c (92%)	13	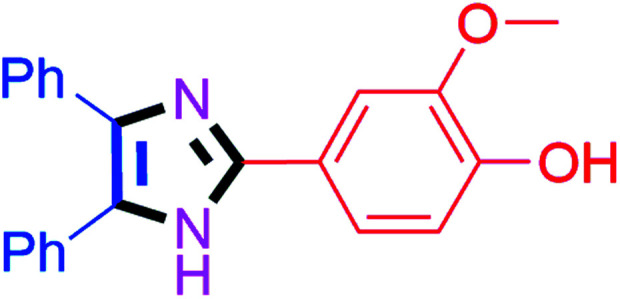	40	4m (90%)
4	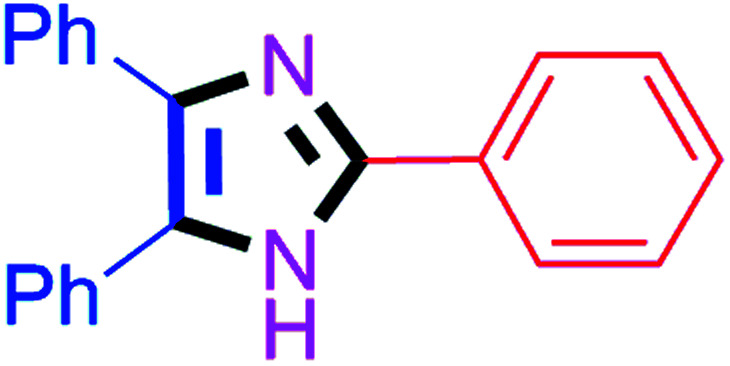	35	4d (86%)	14	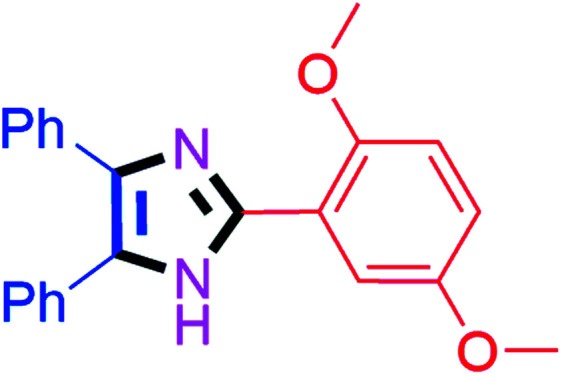	30	4n (92%)
5	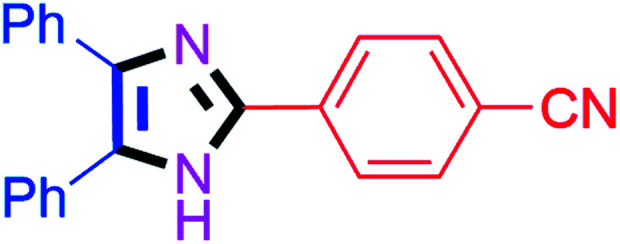	35	4e (84%)	15	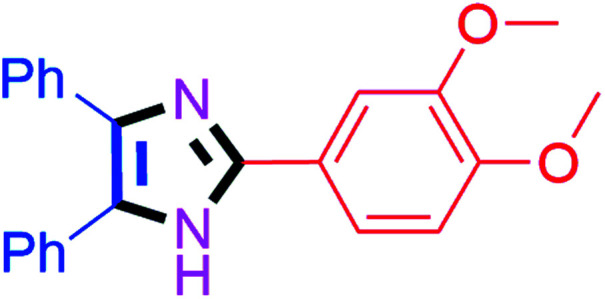	30	4o (94%)
6	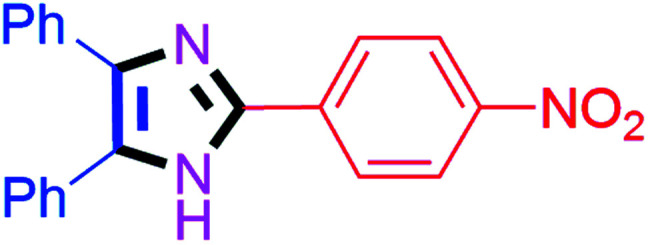	40	4f (92%)	16	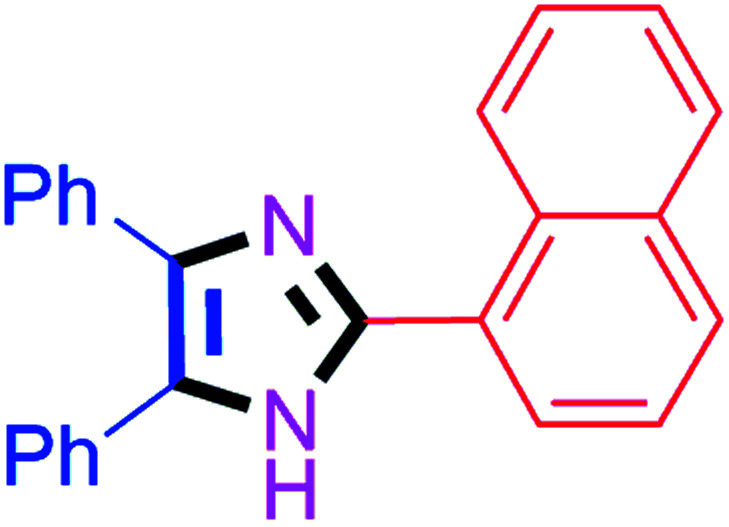	30	4p (95%)
7	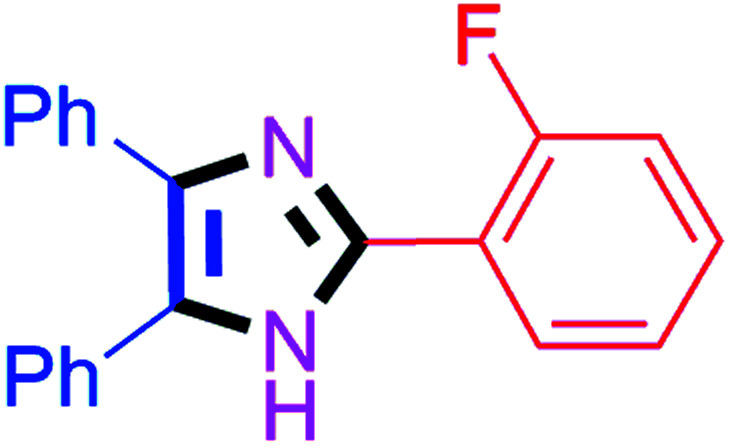	45	4g (89%)	17	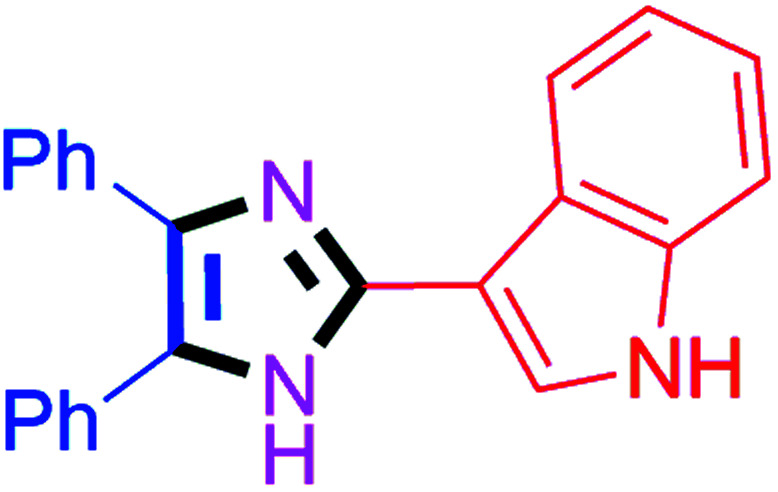	65	4q (84%)
8	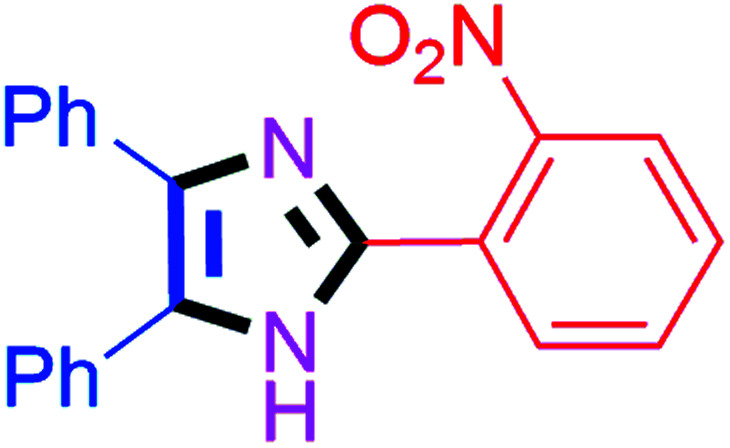	40	4h (95%)	18	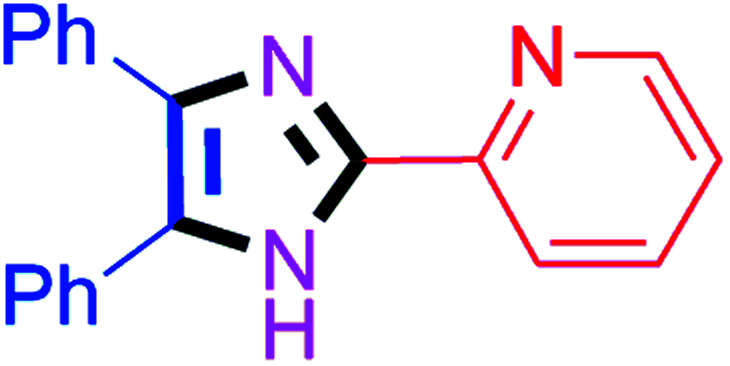	60	4r (83%)
9	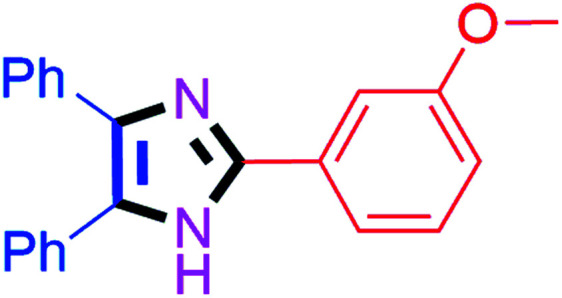	30	4i (92%)	19	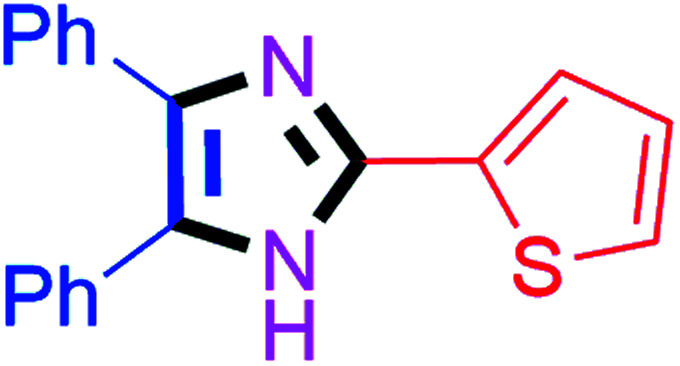	60	4s (88%)
10	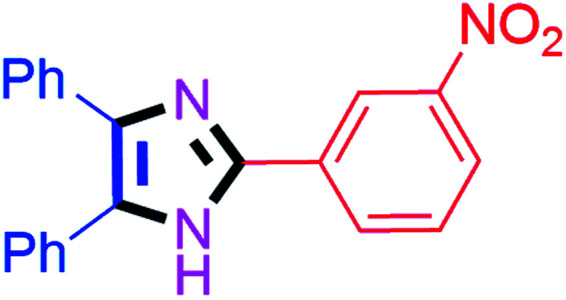	45	4j (96%)	20	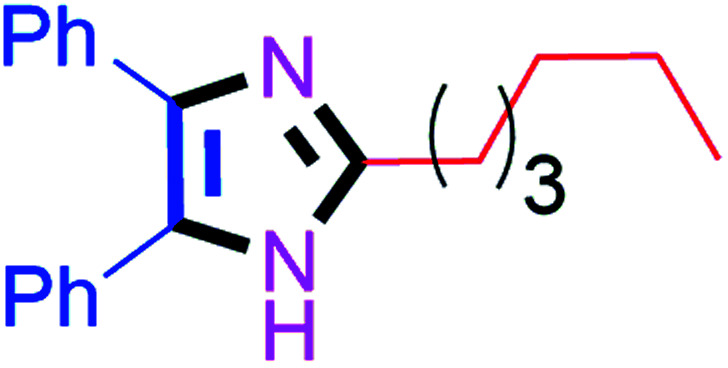	90	4t (80%)

a Reaction conditions: 1 (1 mmol, 1.0 equiv.), 2a (1 mmol, 1.0 equiv.), 3 (3 mmol, 3.0 equiv.), CuI (15 mol%), BuOH (7 mL) at reflux.

b Isolated yield.

Further, the utility of the present method was investigated with aliphatic heptanal aldehyde, where the product 4t was obtained in 80% yield (Table 2, entry 20). With the same optimized conditions, the applicability of the developed system was evaluated using benzil 2b which reacted in the same fashion with different substituted aldehydes and afforded trisubstituted imidazoles in high yields as summarized in Table 3. Various toxic oxidant catalysed reactions are used to synthesise 1,2-diketones such as benzil from α-hydroxy ketone (benzoin).^37–39^ Numerous reports of the synthesis of trisubstituted imidazoles using 1,2-diketones have been reported but not from α-hydroxy ketone. Herein, our developed method for the synthesis of trisubstituted imidazoles from α-hydroxy ketone (benzoin) as well as 1,2-diketones (benzil) has great utility.

**Table 3:** Substrate scope with benzil for trisubstituted imidazole synthesis^a^

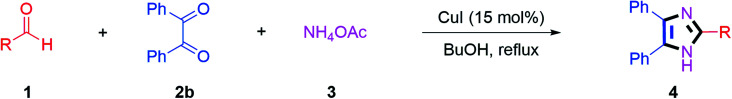							
Entry	4	Time (min)	Yield^b^ (%)	Entry	4	Time (min)	Yield^b^ (%)
1	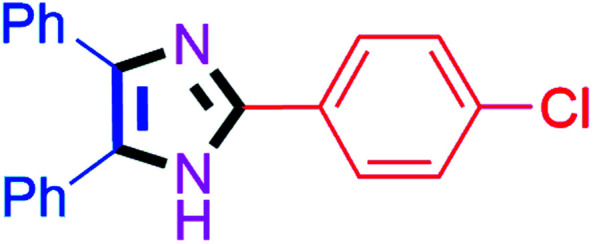	45	4a (84%)	11	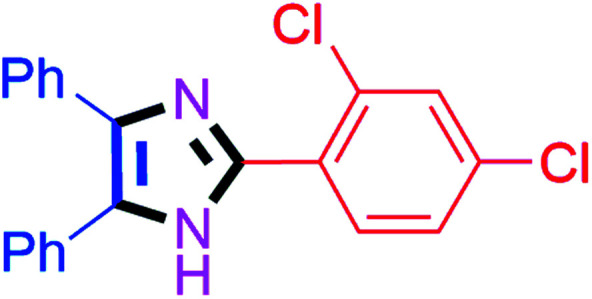	60	4k (91%)
2	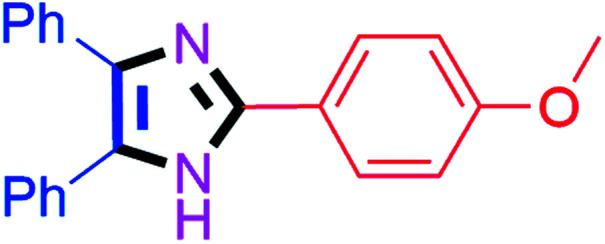	45	4b (92%)	12	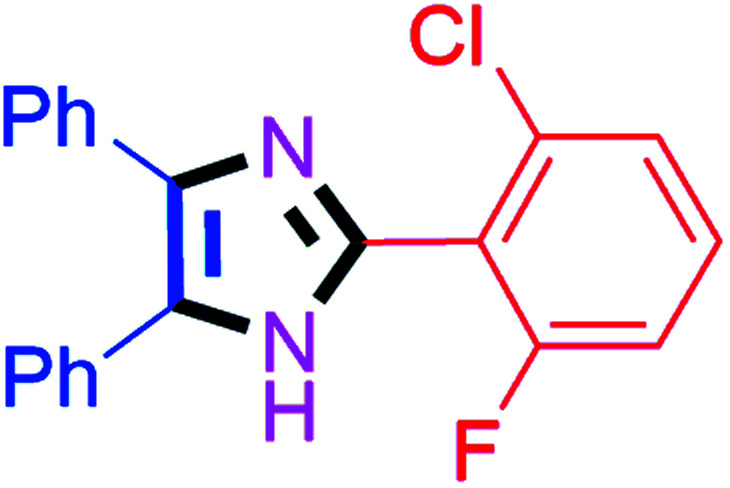	55	4l (92%)
3	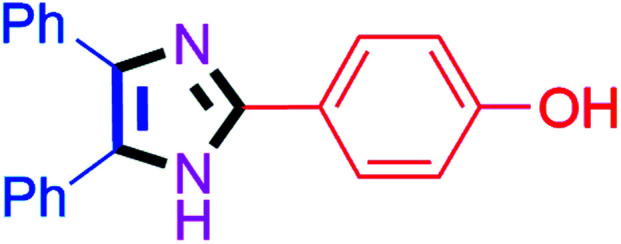	50	4c (91%)	13	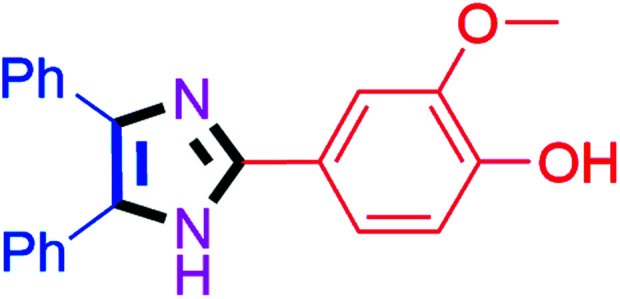	50	4m (88%)
4	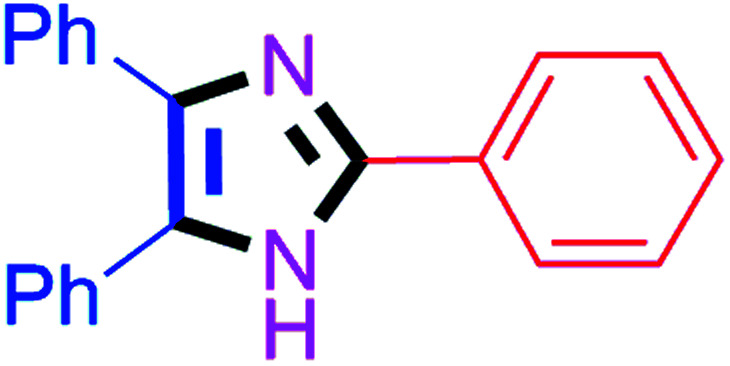	50	4d (85%)	14	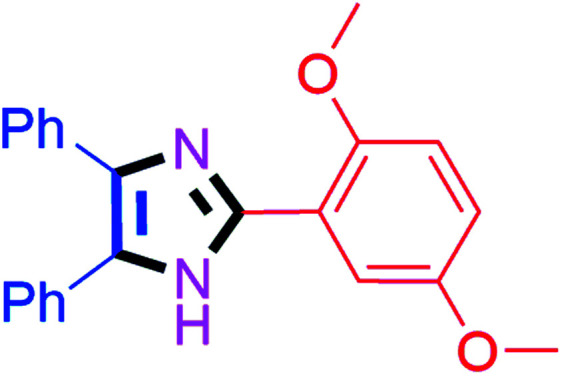	45	4n (90%)
5	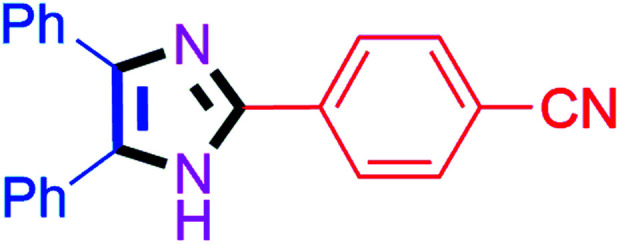	50	4e (82%)	15	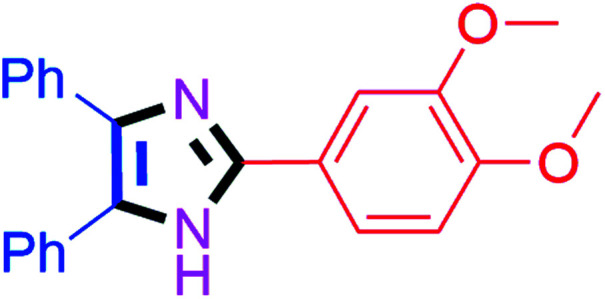	45	4o (93%)
6	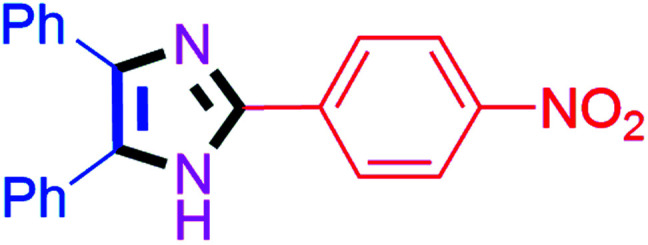	55	4f (91%)	16	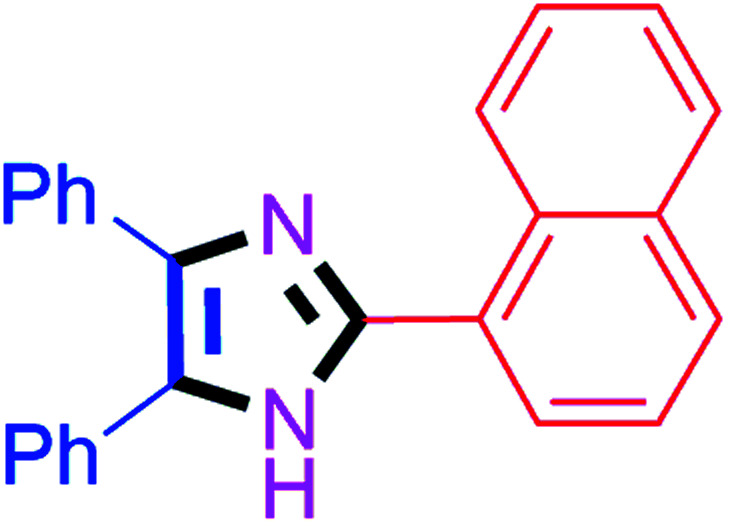	45	4p (94%)
7	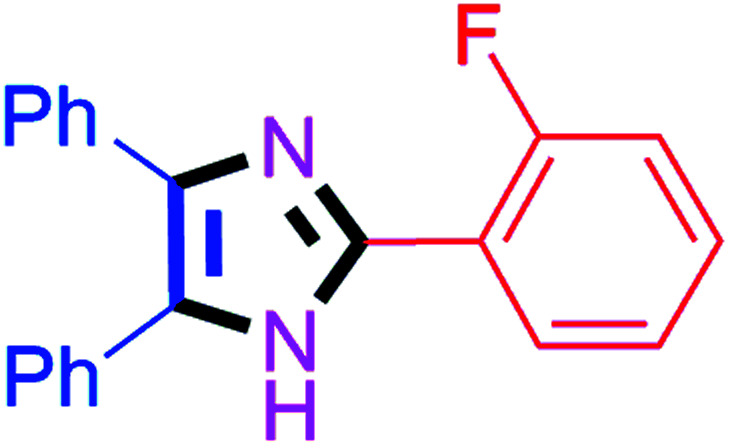	60	4g (88%)	17	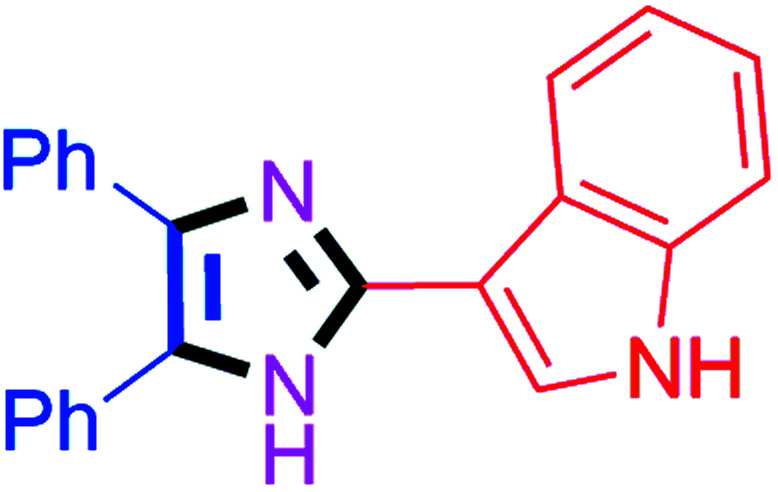	80	4q (83%)
8	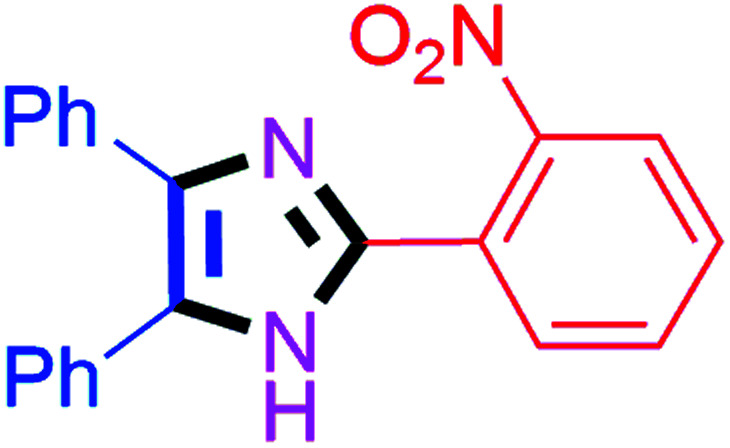	60	4h (94%)	18	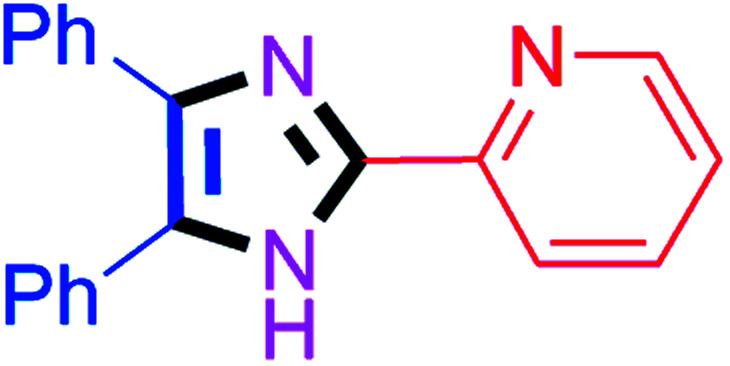	80	4r (81%)
9	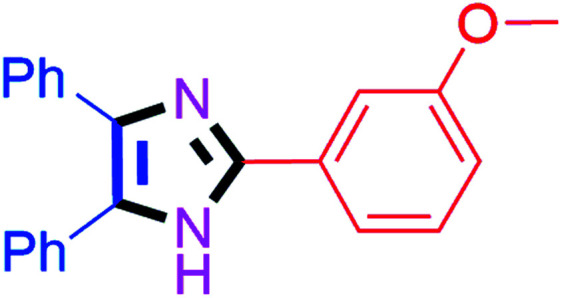	50	4i (90%)	19	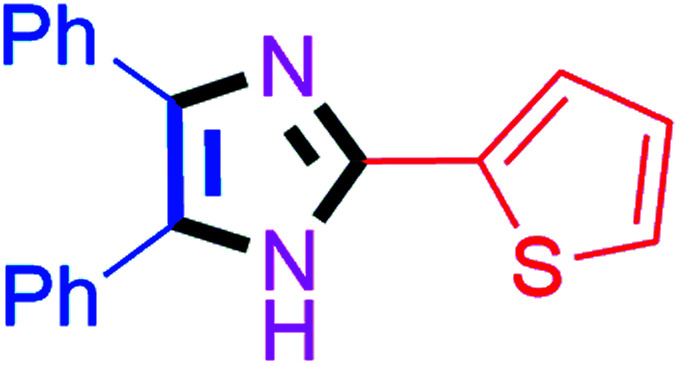	80	4s (87%)
10	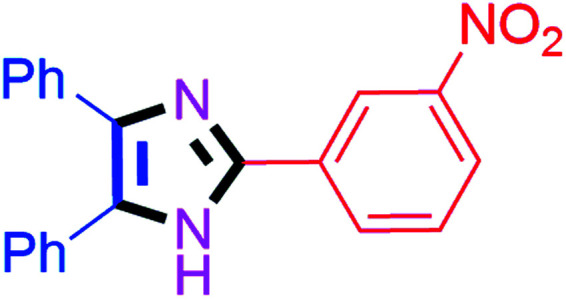	60	4j (94%)	20	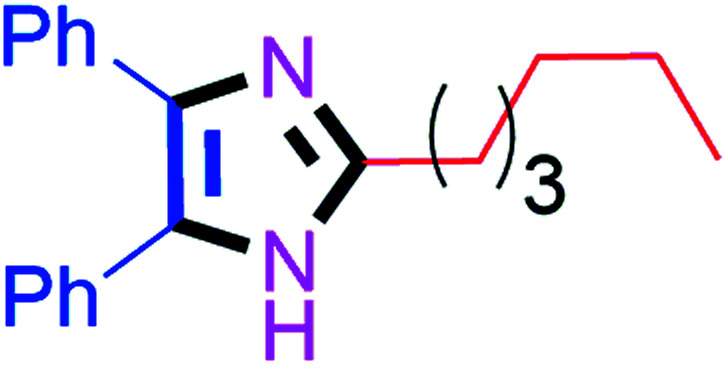	90	4t (79%)

a Reaction conditions: 1 (1 mmol, 1.0 equiv.), 2b (1 mmol, 1.0 equiv.), 3 (3 mmol, 3.0 equiv.), CuI (15 mol%), BuOH (7 mL) at reflux.

b Isolated yield.

**Scheme 1:**
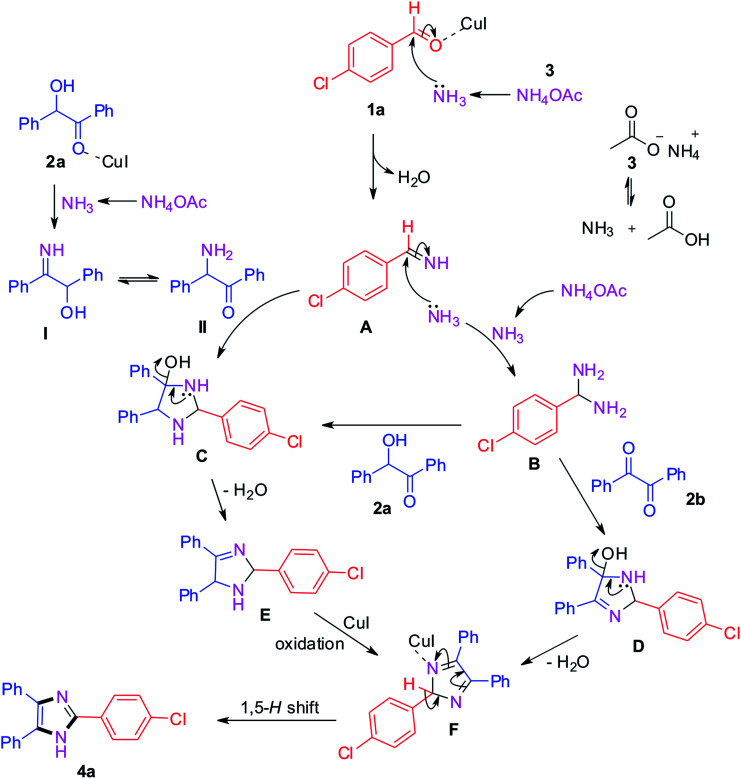
Plausible reaction mechanism.

The plausible mechanism for the trisubstituted imidazole synthesis is summarized in Scheme 1. From the relevant literature and experimental results, different pathways have been proposed for benzil and benzoin as starting materials.^40–45^ Initially, the condensation of 4-chlorobenzaldehyde with ammonia produced from ammonium acetate generates intermediate A.^42,44^ Simultaneously, benzoin 2a reacts with ammonium acetate and leads to the formation of intermediate I which further converts into II*via* tautomerism. Afterwards, the formed intermediates I and II react with intermediate A which converts into intermediate C.^44^ On the other hand, the intermediate A gets converted into (4-chlorophenyl)methanediamine B by reacting with another ammonia molecule. Then, the intermediate B reacts with benzoin 2a and benzil 2b to afford intermediates C and D respectively. After that, intermediate C gets converted into E by loss of water which further converts into intermediate F*via* oxidation in the presence of CuI catalyst.^43–48^ The same F intermediate formation takes place from intermediate D by loss of water. At the end, the desired product 4a formation takes place from common intermediate F*via* [1,5]-H-transfer.^49^

## Conclusion

In conclusion, we have developed a practical method for synthesis of 2,4,5-trisubstituted imidazoles. The present method has a great advantage of substrate scope with α-hydroxy ketone (benzoin) as well as 1,2-diketone (benzil). This three component reaction is effective due to good yields in a short time and simple reaction conditions.

## Experimental section

### General information

All solvents used in the reactions were distilled for purity. The reagents were commercially available and used directly without purification unless otherwise stated. Melting points were recorded with a Thomas-Hoover capillary melting point apparatus. The thin layer chromatography (TLC) was performed using silica gel plates (GF254) with 100–120 mesh size and visualization was effected with a short-wavelength UV lamp (254 nm). ^1^H and ^13^C NMR spectra were recorded using Jeol as well as Bruker NMR instruments at 400 and 100 MHz using DMSO and CDCl_3_ solvents. The chemical shifts are expressed in *δ*, parts per million (ppm) with reference to internal standard TMS. The spin multiplicities are represented by the symbols s (singlet), d (doublet), t (triplet), q (quartet), p (pentet), and m (multiplet) and coupling constants (*J*) are reported in hertz (Hz).

### General procedure for synthesis of imidazoles (4)

In a round bottom flask, the mixture of aldehyde 1 (1 mmol), benzoin 2a or benzil 2b (1 mmol), NH_4_OAc (3 mmol), CuI (15 mol%) and BuOH (7 mL) was refluxed. The progress of the reaction was monitored using thin layer chromatography (TLC) technique. After completion of the reaction, the mixture was cooled to room temperature and poured into crushed ice. The precipitated solid product was stirred at room temperature and filtered to obtain sufficient pure crude product 4. The product was recrystallized by using ethanol solvent.

### Characterization data of pure polysubstituted imidazole products

#### 2-(4-Chlorophenyl)-4,5-diphenyl-1*H*-imidazole (4a)

White solid; mp: 260–262 °C [Lit.^50^ 260–262 °C]; ^1^H NMR (400 MHz, DMSO-d_6_) *δ* 12.87 (s, 1H), 8.06 (dt, *J* = 9.2 and 2.3 Hz, 2H), 7.53–7.32 (m, 9H), 7.28–7.17 (m, 3H) ppm; ^13^C NMR (100 MHz, DMSO-d_6_) *δ* 144.9, 137.8, 135.5, 133.3, 131.4, 129.7, 129.3, 129.2, 129.1, 129.0, 128.8, 128.4, 127.6, 127.4, 127.2 ppm.

#### 2-(4-Methoxyphenyl)-4,5-diphenyl-1*H*-imidazole (4b)

White solid; mp: 228–230 °C [Lit.^50^ 227–229 °C]; ^1^H NMR (400 MHz, DMSO-d_6_) *δ* 12.48 (s, 1H), 7.98 (d, *J* = 9.2 Hz, 2H), 7.51–7.38 (m, 6H), 7.34–7.15 (m, 4H), 7.00 (d, *J* = 9.2 Hz, 2H), 3.77 (s, 3H) ppm; ^13^C NMR (100 MHz, DMSO-d_6_) *δ* 159.9, 146.1, 137.3, 135.8, 131.7, 129.2, 128.9, 128.7, 128.2, 127.6, 127.2, 126.9, 123.6, 114.6, 55.7 ppm.

#### 2-(4-Hydroxyphenyl)-4,5-diphenyl-1*H*-imidazole (4c)

White solid; mp: 269–271 °C [Lit.^50^ 270–272 °C]; ^1^H NMR (400 MHz, DMSO-d_6_) *δ* 9.73 (s, 1H), 7.87 (d, *J* = 8.6 Hz, 2H), 7.48 (d, *J* = 7.3 Hz, 4H), 7.19–7.36 (6H), 6.83 (d, *J* = 8.6 Hz, 2H), 3.41 (s, 1H) ppm; ^13^C NMR (100 MHz, DMSO-d_6_) *δ* 158.4, 146.6, 134.0, 133.8, 129.6, 129.3, 128.9, 128.7, 128.2, 127.5, 127.4, 127.0, 126.7, 122.0, 116.0 ppm.

#### 2,4,5-Triphenyl-1*H*-imidazole (4d)

White solid; mp: 276–278 °C [Lit.^50^ 275–277 °C]; ^1^H NMR (400 MHz, CDCl_3_) *δ* 9.26 (s, 1H, NH), 7.94–7.91 (m, 2H), 7.63–7.45 (m, 6H), 7.42–7.01 (m, 7H) ppm.

#### 4-(4,5-Diphenyl-1*H*-imidazol-2-yl)benzonitrile (4e)

White solid; mp: 186–187 °C [Lit.^50^ 185 °C]; ^1^H NMR (400 MHz, DMSO-d_6_) *δ* 12.99 (s, 1H), 8.21–7.90 (m, 4H), 7.49–7.27 (m, 10H) ppm; ^13^C NMR (100 MHz, DMSO-d_6_) *δ* 144.2, 138.6, 135.2, 134.8, 133.3, 131.1, 130.1, 129.3, 129.1, 128.8, 127.6, 127.4, 126.0, 119.5, 110.6 ppm.

#### 2-(4-Nitrophenyl)-4,5-diphenyl-1*H*-imidazole (4f)

Orange solid; mp: 242–244 °C [Lit.^51^ 230–232 °C]; ^1^H NMR (400 MHz, DMSO-d_6_) *δ* 13.12 (s, 1H), 8.35–8.25 (m, 4H), 7.62–7.35 (m, 8H), 7.30–7.20 (m, 2H) ppm; ^13^C NMR (100 MHz, DMSO-d_6_) *δ* 147.0, 143.9, 139.0, 136.6, 135.1, 131.0, 130.6, 129.6, 129.3, 129.1, 128.8, 127.7, 127.5, 126.3, 124.8 ppm.

#### 2-(2-Fluorophenyl)-4,5-diphenyl-1*H*-imidazole (4g)

White solid; mp: 257–259 °C [Lit.^50^ 188–189 °C]; ^1^H NMR (400 MHz, DMSO-d_6_) *δ* 12.53 (s, 1H), 7.96 (t, *J* = 7.0 Hz, 1H), 7.51–7.25 (m, 12H), 7.19 (t, *J* = 7.3 Hz, 1H) ppm; ^13^C NMR (100 MHz, DMSO-d_6_) *δ* 160.6, 158.1, 141.4, 137.7, 135.5, 131.4, 131.0, 130.9, 130.2, 129.2, 129.1, 128.7, 128.4, 127.7, 127.1, 125.2, 119.3, 119.1, 116.9, 116.7 ppm.

#### 2-(2-Nitrophenyl)-4,5-diphenyl-1*H*-imidazole (4h)

Yellow solid; mp: 246–250 °C [Lit.^52^ 247–249 °C]; ^1^H NMR (400 MHz, DMSO-d_6_) *δ* 12.95 (s, 1H), 7.96 (d, *J* = 7.9 Hz, 1H), 7.89 (d, *J* = 7.9 Hz, 1H), 7.75 (t, *J* = 7.6 Hz, 1H), 7.65–7.58 (m, 1H), 7.48–7.33 (m, 7H), 7.27 (t, *J* = 7.3 Hz, 2H), 7.19 (t, *J* = 7.3 Hz, 1H) ppm; ^13^C NMR (100 MHz, DMSO-d_6_) *δ* 148.9, 141.6, 138.1, 135.2, 132.7, 131.2, 130.3, 130.1, 129.3, 129.3, 128.8, 128.6, 127.5, 127.3, 124.6, 123.9 ppm.

#### 2-(3-Methoxyphenyl)-4,5-diphenyl-1*H*-imidazole (4i)

White solid; mp: 290–295 °C [Lit.^53^ 266–268 °C]; ^1^H NMR (400 MHz, DMSO-d_6_) *δ* 12.66 (s, 1H), 7.65–7.62 (m, 2H), 7.52–7.33 (m, 8H), 7.27 (t, *J* = 7.6 Hz, 2H), 7.18 (t, *J* = 7.0 Hz, 1H), 6.91 (dd, *J* = 7.6, 2.1 Hz, 1H), 3.79 (s, 3H) ppm; ^13^C NMR (100 MHz, DMSO-d_6_) *δ* 160.1, 145.9, 137.6, 135.6, 132.1, 131.6, 130.4, 129.2, 129.0, 128.8, 128.7, 128.4, 127.6, 127.1, 118.1, 114.8, 110.7, 55.7 ppm.

#### 2-(3-Nitrophenyl)-4,5-diphenyl-1*H*-imidazole (4j)

Yellow solid; mp: 226–228 °C [Lit.^50^ 230 °C]; ^1^H NMR (400 MHz, CDCl_3_) *δ* 12.49 (s, 1H, NH), 9.02 (s, 1H), 8.53 (d, *J* = 7.2 Hz, 1H), 8.11 (d, *J* = 6.4 Hz, 1H), 7.61–7.51 (m, 6H), 7.35–7.19 (m, 5H) ppm.

#### 2-(2,4-Dichlorophenyl)-4,5-diphenyl-1*H*-imidazole (4k)

White solid; mp: 174–176 °C [Lit.^54^ 174–175 °C]; ^1^H NMR (400 MHz, DMSO-d_6_) *δ* 12.64 (s, 1H), 7.81 (dd, *J* = 8.9, 6.4 Hz, 1H), 7.59 (dd, *J* = 8.6, 2.4 Hz, 1H), 7.51–7.17 (m, 11H) ppm; ^13^C NMR (100 MHz, DMSO-d_6_) *δ* 143.1, 137.4, 135.5, 133.7, 133.6, 131.4, 129.2, 128.8, 128.7, 128.6, 128.3, 127.7, 127.1, 118.1, 117.9, 115.3, 115.1 ppm.

#### 2-(2-Chloro-6-fluorophenyl)-4,5-diphenyl-1*H*-imidazole (4l)

White solid; mp: 203–205 °C [Lit.^55^ 204–206 °C]; ^1^H NMR (400 MHz, DMSO-d_6_) *δ* 12.86 (s, 1H), 7.65–7.46 (m, 6H), 7.40–7.22 (m, 7H) ppm; ^13^C NMR (100 MHz, DMSO-d_6_) *δ* 162.6, 160.1, 137.9, 135.2, 135.1, 132.5, 132.4, 129.3, 128.8, 128.4, 127.7, 126.3, 126.3, 120.3, 120.1, 115.5, 115.2 ppm.

#### 4-(4,5-Diphenyl-1*H*-imidazol-2-yl)-2-methoxyphenol (4m)

Off white solid; mp: 259–261 °C [Lit.^50^ 260 °C]; ^1^H NMR (400 MHz, DMSO-d_6_) *δ* 12.40 (s, 1H), 9.26 (s, 1H), 7.60 (d, *J* = 1.8 Hz, 1H), 7.51–7.30 (m, 8H), 7.34–7.15 (m, 3H), 6.82 (d, *J* = 7.9 Hz, 1H), 3.82 (s, 3H) ppm; ^13^C NMR (100 MHz, DMSO-d_6_) *δ* 148.2, 147.5, 146.6, 137.1, 135.9, 131.9, 129.2, 128.9, 128.7, 128.1, 127.9, 127.5, 126.9, 122.4, 118.8, 116.1, 109.8, 56.2 ppm.

#### 2-(2,5-Dimethoxyphenyl)-4,5-diphenyl-1*H*-imidazole (4n)

White solid; mp: 180–182 °C [Lit.^56^ 185–186 °C]; ^1^H NMR (400 MHz, CDCl_3_) *δ* 10.6 (s, 1H, NH), 8.03 (d, *J* = 3.2 Hz, 1H), 7.69–6.95 (m, 10H), 6.96 (d, *J* = 8.8 Hz, 1H), 6.89 (dd, *J* = 9.2 & 3.2 Hz, 1H), 3.99 (s, 3H, OMe), 3.89 (s, 3H. OMe) ppm; ^13^C NMR (100 MHz, CDCl_3_) *δ* 154.3, 150.2, 144.0, 128.9, 128.3, 127.8, 116.2, 112.7, 112.1, 56.4, 56.0 ppm.

#### 2-(3,4-Dimethoxyphenyl)-4,5-diphenyl-1*H*-imidazole (4o)

White solid; mp: 218–220 °C [Lit.^50^ 220 °C]; ^1^H NMR (400 MHz, CDCl_3_) *δ* 7.58–7.47 (m, 5H), 7.37–7.24 (m, 8H), 6.90 (d, *J* = 7.2 Hz, 1H), 3.92 (s, 3H, OMe), 3.91 (s, 3H, OMe) ppm; ^13^C NMR (100 MHz, CDCl_3_) *δ* 149.8, 149.4, 146.1, 127.8, 127.4, 123.1, 117.4, 111.1, 109.0, 56.0, 55.9 ppm.

#### 2-(Naphthalen-1-yl)-4,5-diphenyl-1*H*-imidazole (4p)

White solid; mp: 263–265 °C [Lit.^57^ 262–263 °C]; ^1^H NMR (400 MHz, DMSO-d_6_) *δ* 12.77 (s, 1H), 9.15 (d, *J* = 8.6 Hz, 1H), 7.97 (d, *J* = 7.9 Hz, 2H), 7.93 (d, *J* = 6.7 Hz, 1H), 7.61–7.52 (m, 7H), 7.43–7.20 (m, 6H) ppm; ^13^C NMR (100 MHz, DMSO-d_6_) *δ* 146.1, 137.6, 135.8, 134.2, 131.5, 130.8, 129.5, 129.2, 128.9, 128.8, 128.5, 128.3, 127.9, 127.7, 127.3, 127.1, 126.7, 125.8 ppm.

#### 3-(4,5-Diphenyl-1*H*-imidazol-2-yl)-1*H*-indole (4q)

Off white solid; mp: 307–308 °C [Lit.^58^ 308–310 °C]; ^1^H NMR (400 MHz, DMSO-d_6_) *δ* 12.25 (s, 1H), 11.34 (d, *J* = 1.8 Hz, 1H), 8.44 (d, *J* = 7.3 Hz, 1H), 8.18–7.96 (m, 1H), 7.67–7.54 (m, 2H), 7.49–7.26 (m, 8H), 7.19–7.09 (m, 3H) ppm; ^13^C NMR (100 MHz, DMSO-d_6_) *δ* 144.2, 136.8, 136.5, 136.2, 132.1, 129.2, 128.7, 127.9, 127.4, 126.7, 126.3, 125.6, 124.3, 122.4, 122.0, 120.2, 112.1, 107.3 ppm.

#### 2-(4,5-Diphenyl-1*H*-imidazol-2-yl)pyridine (4r)

Greenish yellow solid; mp: 173–175 °C [Lit.^59^ 174–176 °C]; ^1^H NMR (400 MHz, DMSO-d_6_) *δ* 13.15 (s, 1H), 8.60 (s, 1H), 8.12–7.87 (m, 2H), 7.64–7.46 (m, 5H), 7.35–7.19 (m, 6H) ppm; ^13^C NMR (100 MHz, DMSO-d_6_) *δ* 149.5, 149.3, 146.0, 138.4, 137.8, 135.6, 131.2, 129.8, 129.1, 129.0, 128.7, 128.3, 127.9, 127.2, 123.7, 120.4 ppm.

#### 4,5-Diphenyl-2-(thiophen-2-yl)-1*H*-imidazole (4s)

Faint yellow solid; mp: 262–264 °C [Lit.^55^ 259–261 °C]; ^1^H NMR (400 MHz, DMSO-d_6_) *δ* 12.77 (s, 1H), 7.66 (d, *J* = 3.1 Hz, 1H), 7.52–7.31 (m, 8H), 7.26 (t, *J* = 7.6 Hz, 2H), 7.20–7.10 (m, 2H) ppm; ^13^C NMR (100 MHz, DMSO-d_6_) *δ* 142.1, 137.3, 135.3, 134.5, 131.4, 129.3, 128.8, 128.8, 128.5, 128.4, 128.2, 127.6, 127.1, 126.8, 124.8 ppm.

#### 2-Hexyl-4,5-diphenyl-1*H*-imidazole (4t)

Faint yellow solid; mp: 130–132 °C [Lit.^54^ 129–130 °C]; ^1^H NMR (400 MHz, DMSO-d_6_) *δ* 12.07 (s, 1H), 7.39 (d, *J* = 6.7 Hz, 4H), 7.27 (t, *J* = 7.6 Hz, 4H), 7.19 (t, *J* = 7.3 Hz, 2H), 2.60 (t, *J* = 7.9 Hz, 2H), 1.70–1.63 (m, 2H), 1.33–1.25 (m, 6H), 0.83 (t, *J* = 7.0 Hz, 3H) ppm; ^13^C NMR (100 MHz, DMSO-d_6_) *δ* 148.9, 134.3, 134.1, 133.9, 128.9, 128.6, 128.4, 128.1, 127.8, 127.5, 127.2, 31.5, 29.0, 28.6, 28.4, 22.6, 14.5 ppm.

## Conflicts of interest

There are no conflicts of interest to declare.

## Supplementary Material

RA-011-D1RA01767E-s001
