# Supplementary material for: Simple practical method for synthesis of trisubstituted imidazoles: an efficient copper catalyzed multicomponent reaction
Source: RSC Adv. 2021 Jun 22;11(36):21955–63. doi: 10.1039/d1ra01767e (PMC9034145; doi:10.1039/d1ra01767e)

Electronic Supplementary Material (ESI) for RSC Advances.
This journal is © The Royal Society of Chemistry 2021

Supporting Information

Simple Practical Method for Synthesis of Trisubstituted Imidazoles: An Efficient Copper Catalyzed Multicomponent Reaction

Vikas D. Kadu*, Ganesh A. Mali, Siddheshwar P. Khadul, Gokul J. Kothe
[a]School of Chemical Sciences, Punyashlok Ahilyadevi Holkar Solapur University
Solapur- 413255, Maharashtra (India)
*Corresponding Authors: Email- vikaskadu1@gmail.com

Contents

Spectral data of trisubstituted imidazoles 3

1.1 ^{1}H NMR of compound: 4a 3

1.2 ^{13}C NMR of compound: 4a 3

1.3 ^{1}H NMR of compound: 4b 4

1.4 ^{13}C NMR of compound: 4b 4

1.5 ^{1}H NMR of compound: 4c 5

1.6 ^{13}C NMR of compound: 4c 5

1.7 ^{1}H NMR of compound: 4d 6

1.8 ^{1}H NMR of compound: 4e 6

1.9 ^{13}C NMR of compound: 4e 7

1.10 ^{1}H NMR of compound: 4f 7

1.11 ^{13}C NMR of compound: 4f 8

1.12 ^{1}H NMR of compound: 4g 8

1.13 ^{13}C NMR of compound: 4g 9

1.14 ^{1}H NMR of compound: 4h 9

1.15 ^{13}C NMR of compound: 4h 10
1.16 ^{1}H NMR of compound: 4i 10
1.17 ^{13}C NMR of compound: 4i 11
1.18 ^{1}H NMR of compound: 4j 11
1.19 ^{1}H NMR of compound: 4k 12
1.20 ^{13}C NMR of compound: 4k 12
1.21 ^{1}H NMR of compound: 4l 13
1.22 ^{13}C NMR of compound: 4l 13
1.23 ^{1}H NMR of compound: 4m 14
1.24 ^{13}C NMR of compound: 4m 14
1.25 ^{1}H NMR of compound: 4n 15
1.26 ^{13}C NMR of compound: 4n 15
1.27 ^{1}H NMR of compound: 4o 16
1.28 ^{13}C NMR of compound: 4o 16
1.29 ^{1}H NMR of compound: 4p 17
1.30 ^{13}C NMR of compound: 4p 17
1.31 ^{1}H NMR of compound: 4q 18
1.32 ^{13}C NMR of compound: 4q 18
1.33 ^{1}H NMR of compound: 4r 19
1.34 ^{13}C NMR of compound: 4r 19
1.35 ^{1}H NMR of compound: 4s 20
1.36 ^{13}C NMR of compound: 4s 20
1.37 ^{1}H NMR of compound: 4t 21
1.38 ^{13}C NMR of compound: 4t 21

Spectral data of trisubstituted imidazoles

1.1 ^{1}H NMR of compound: **4a**

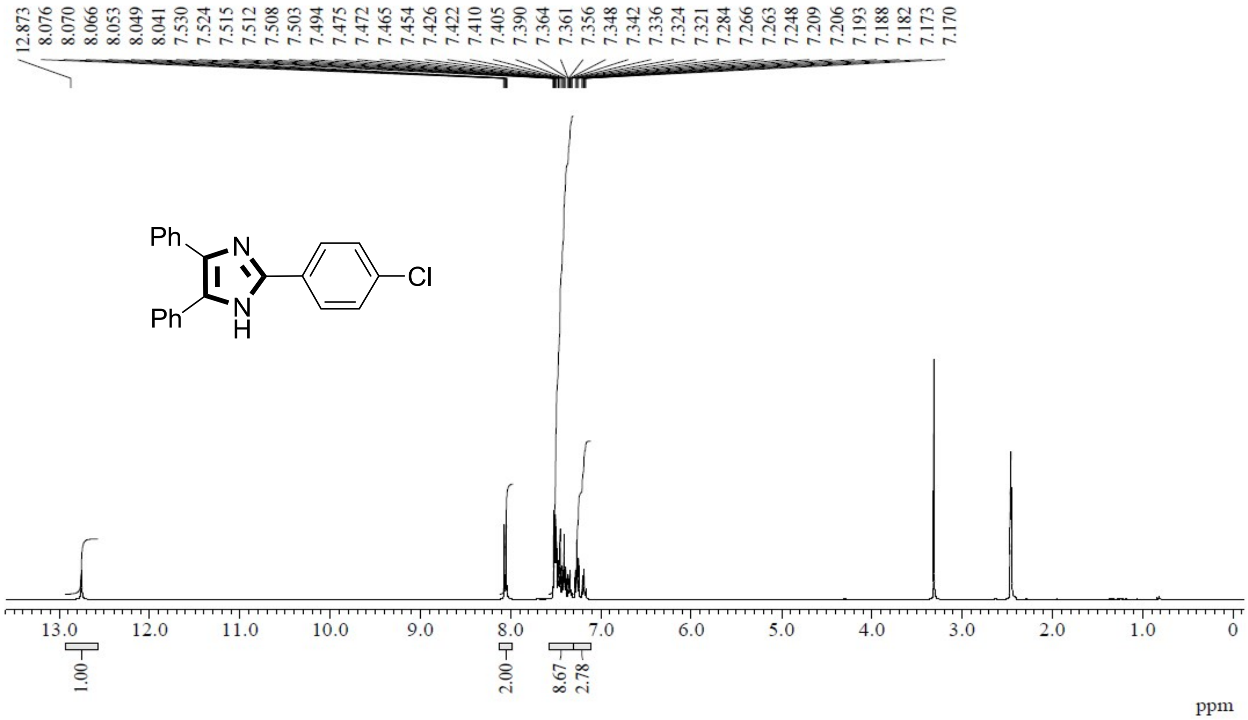

1.2 ^{13}C NMR of compound: **4a**

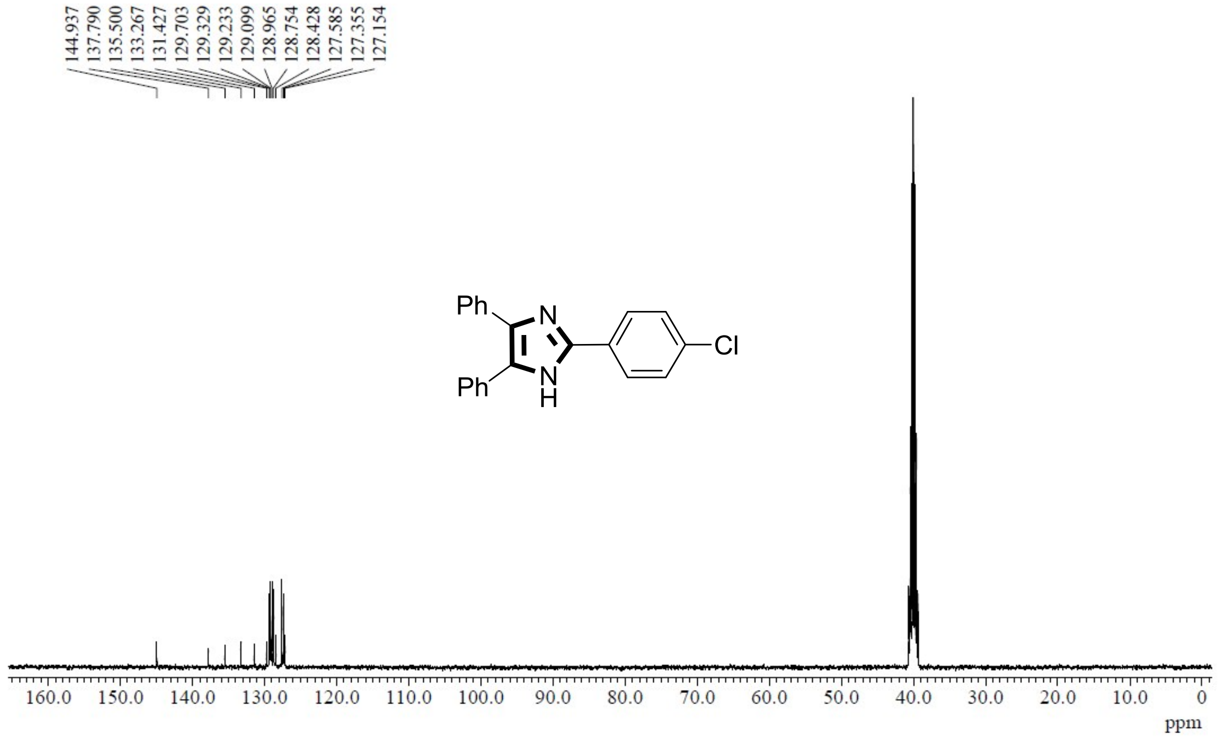

1.3 ^{1}H NMR of compound: **4b**

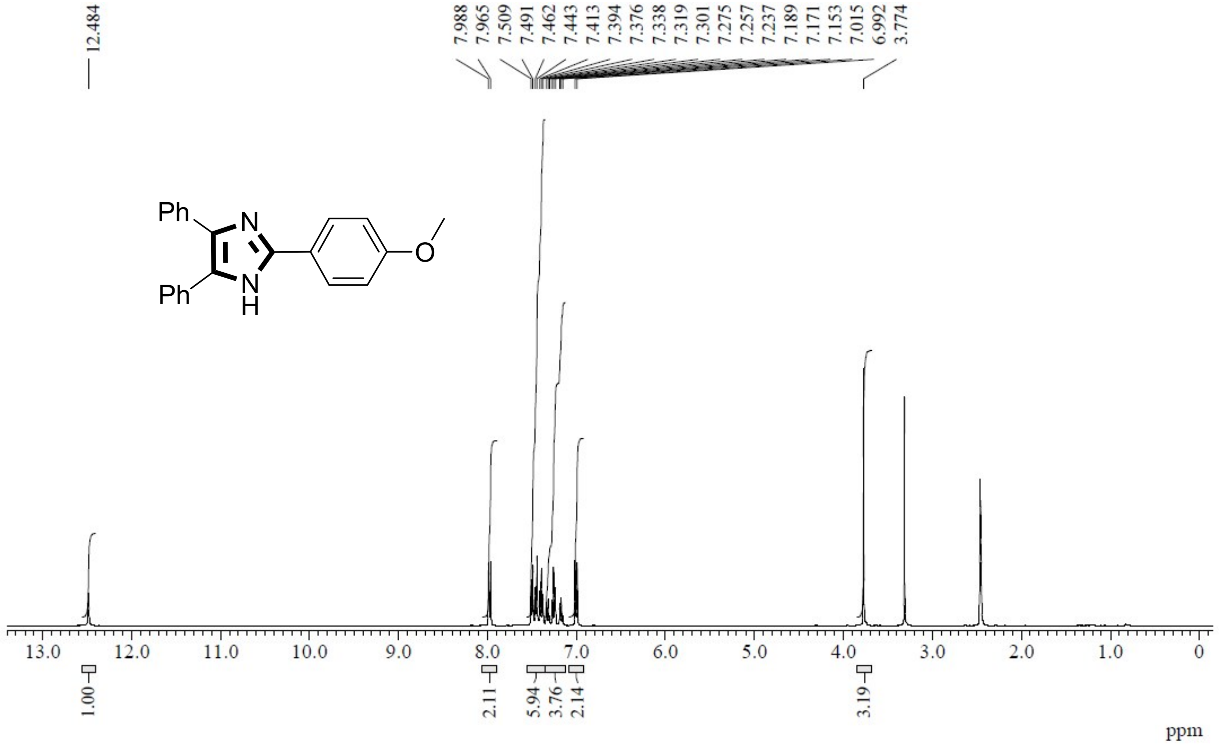

1.4 ^{13}C NMR of compound: **4b**

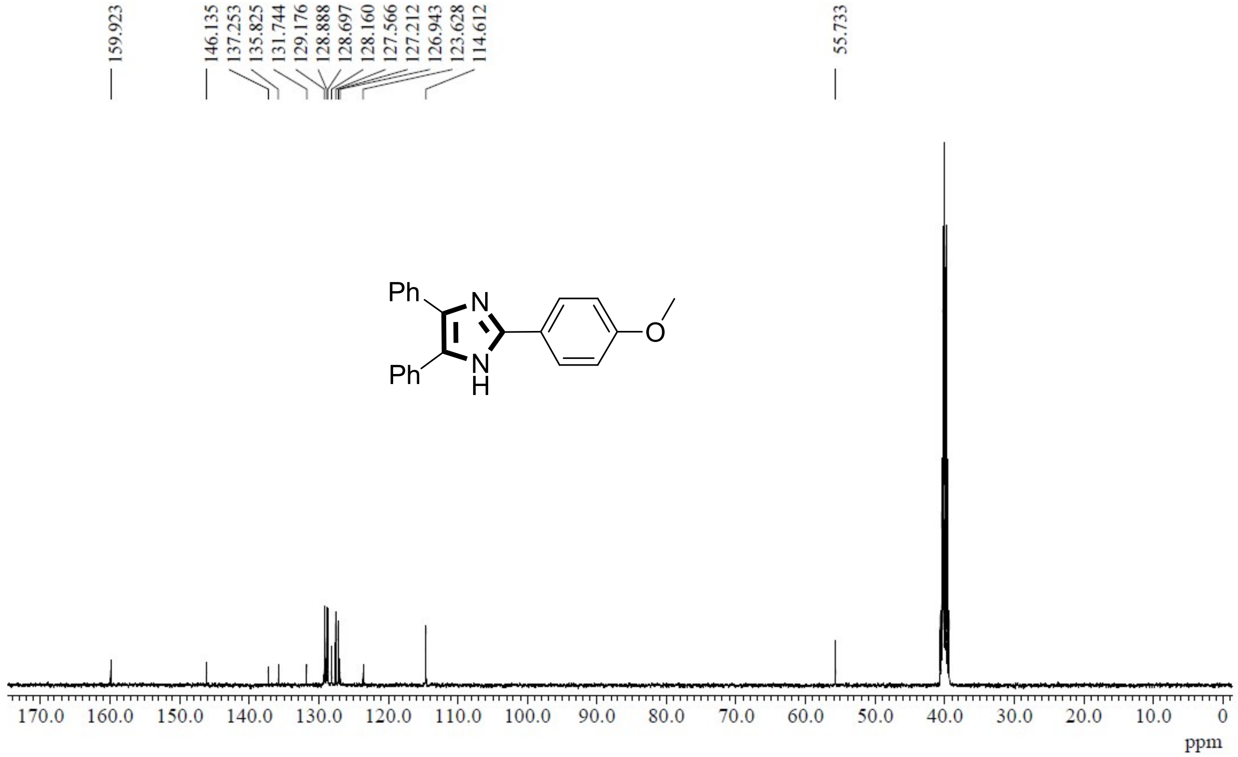

1.5 ^{1}H NMR of compound: **4c**

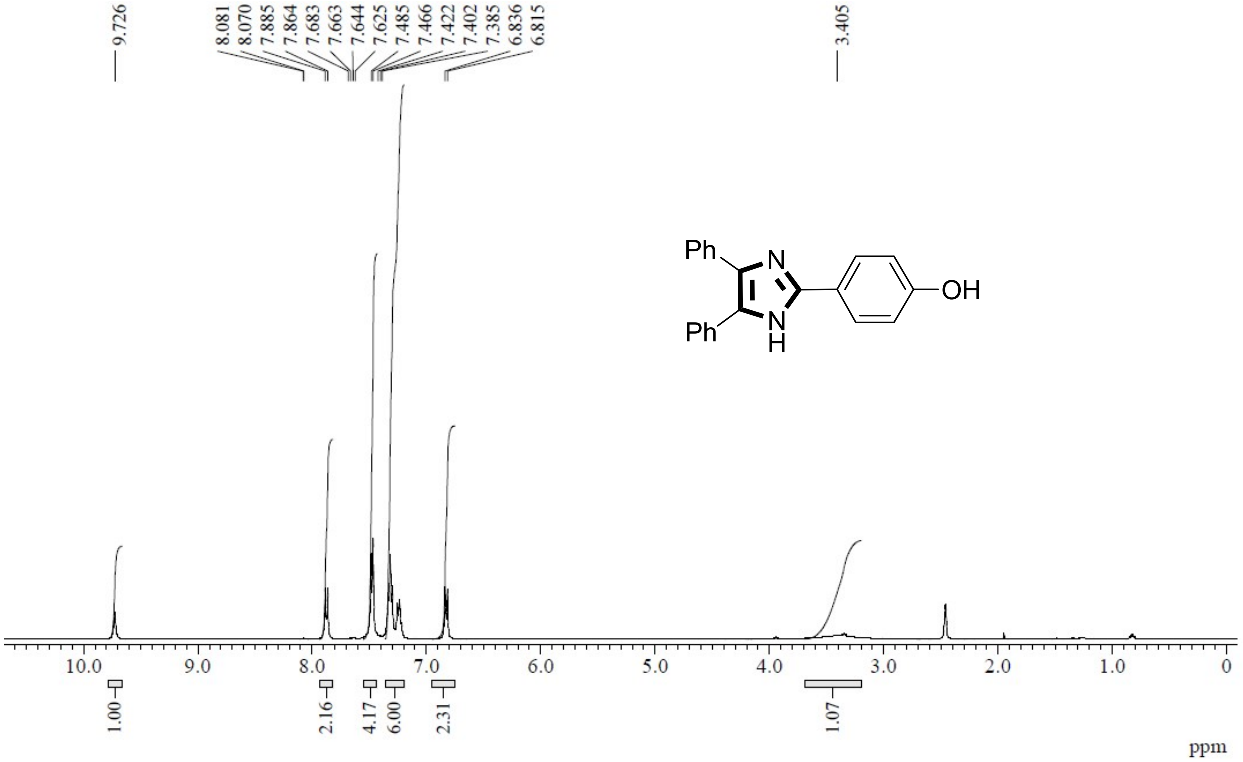

1.6 ^{13}C NMR of compound: **4c**

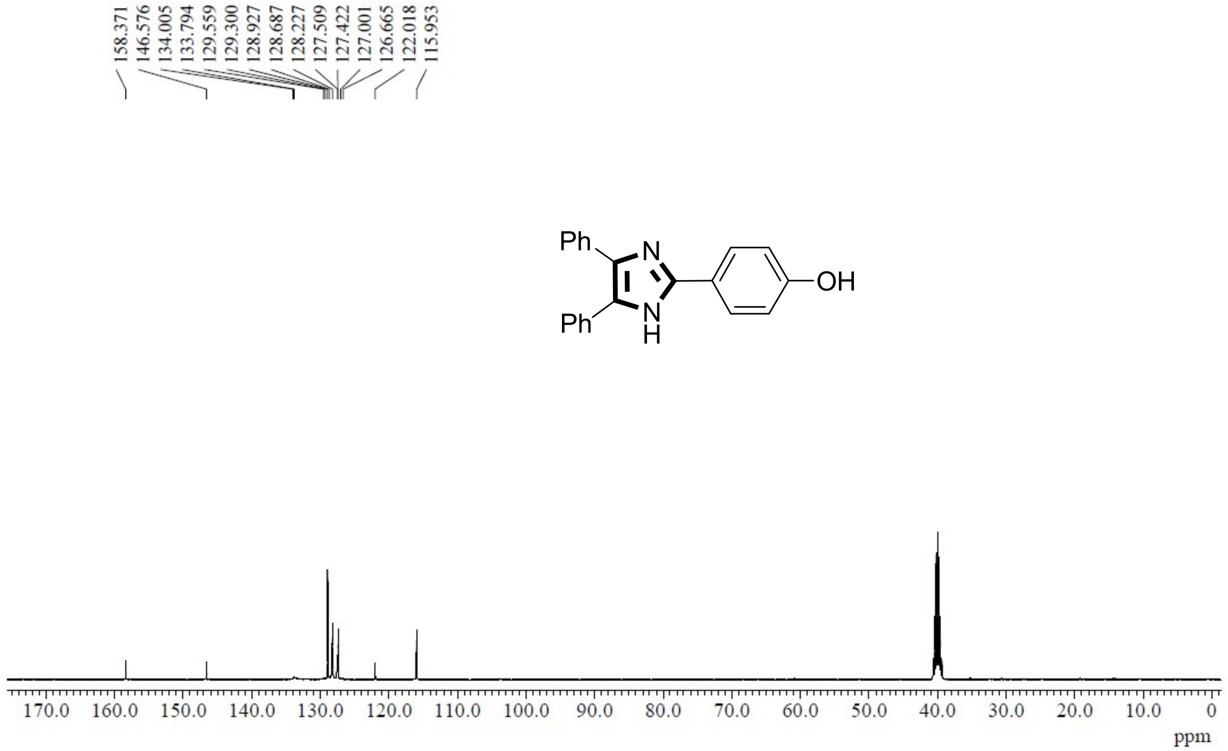

1.7 ^{1}H NMR of compound: **4d**

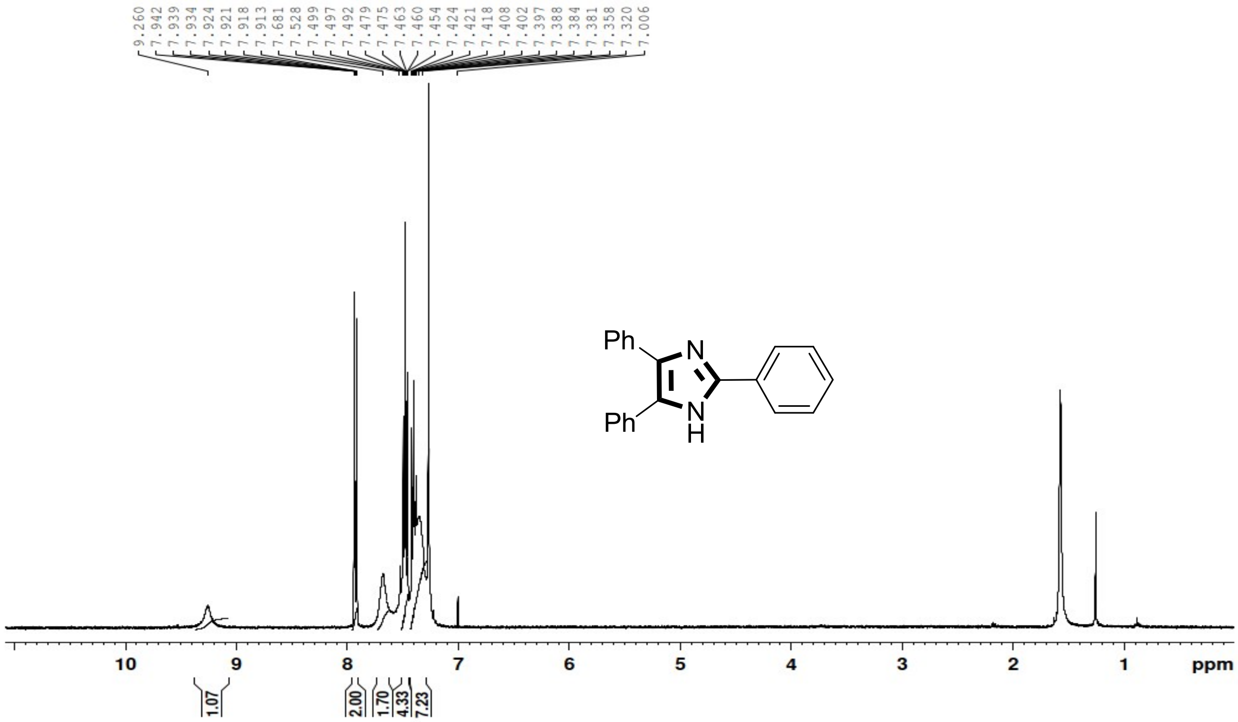

1.8 ^{1}H NMR of compound: **4e**

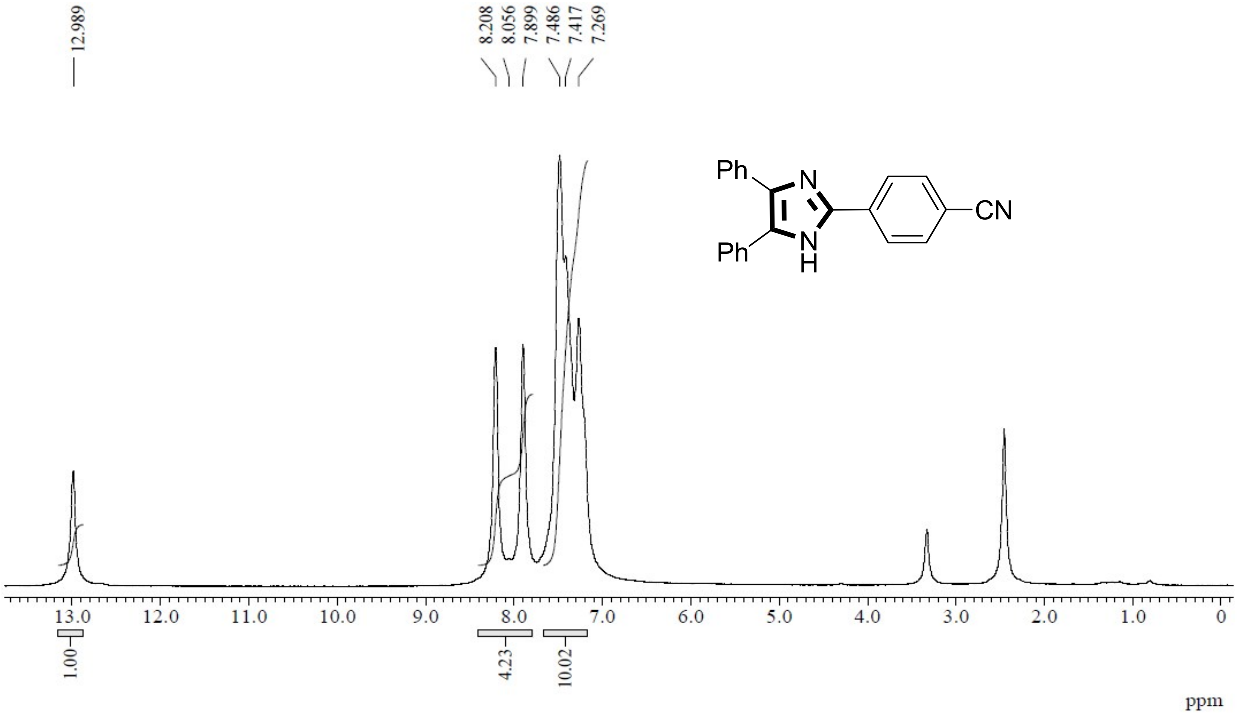

1.9 ^{13}C NMR of compound: **4e**

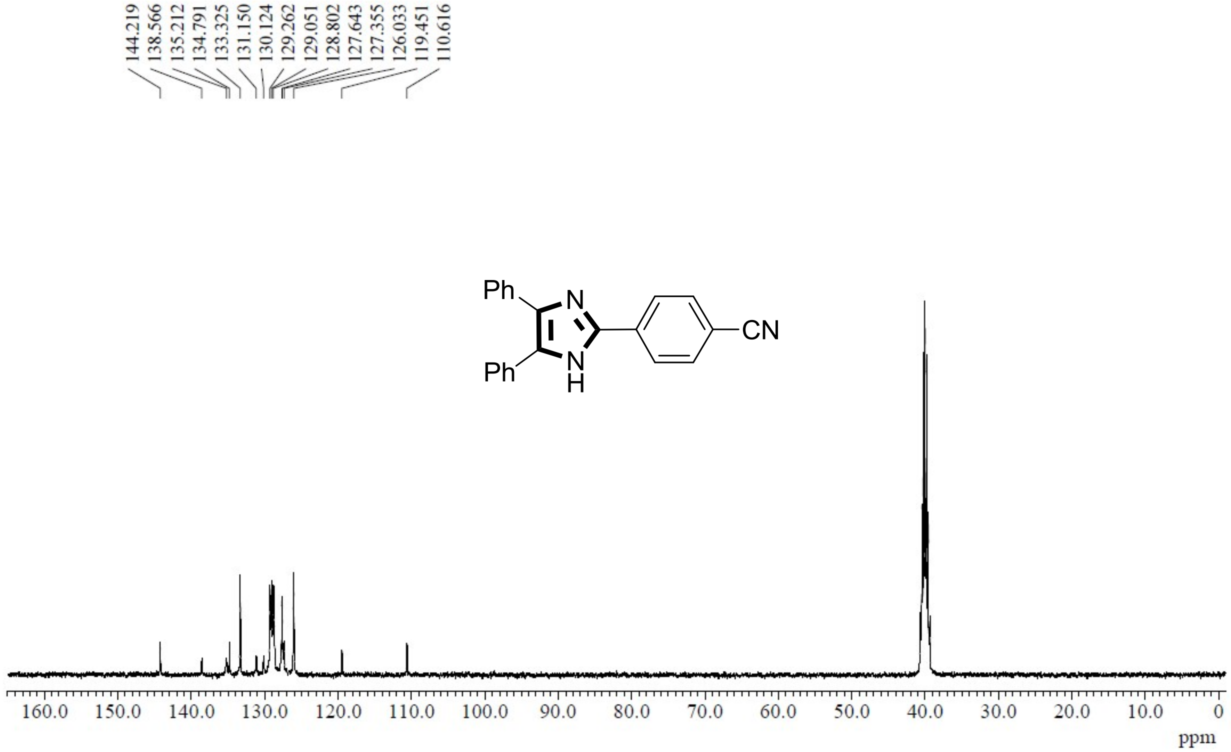

1.10 ^{1}H NMR of compound: **4f**

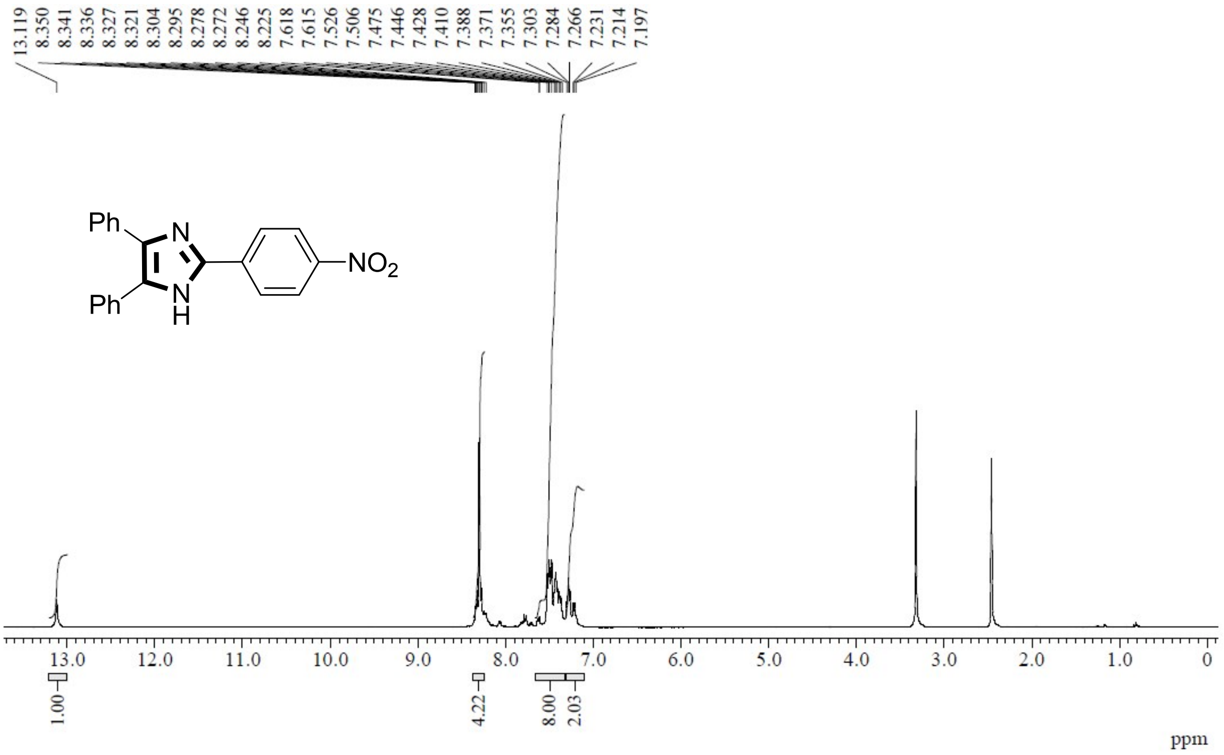

1.11 ^{13}C NMR of compound: **4f**

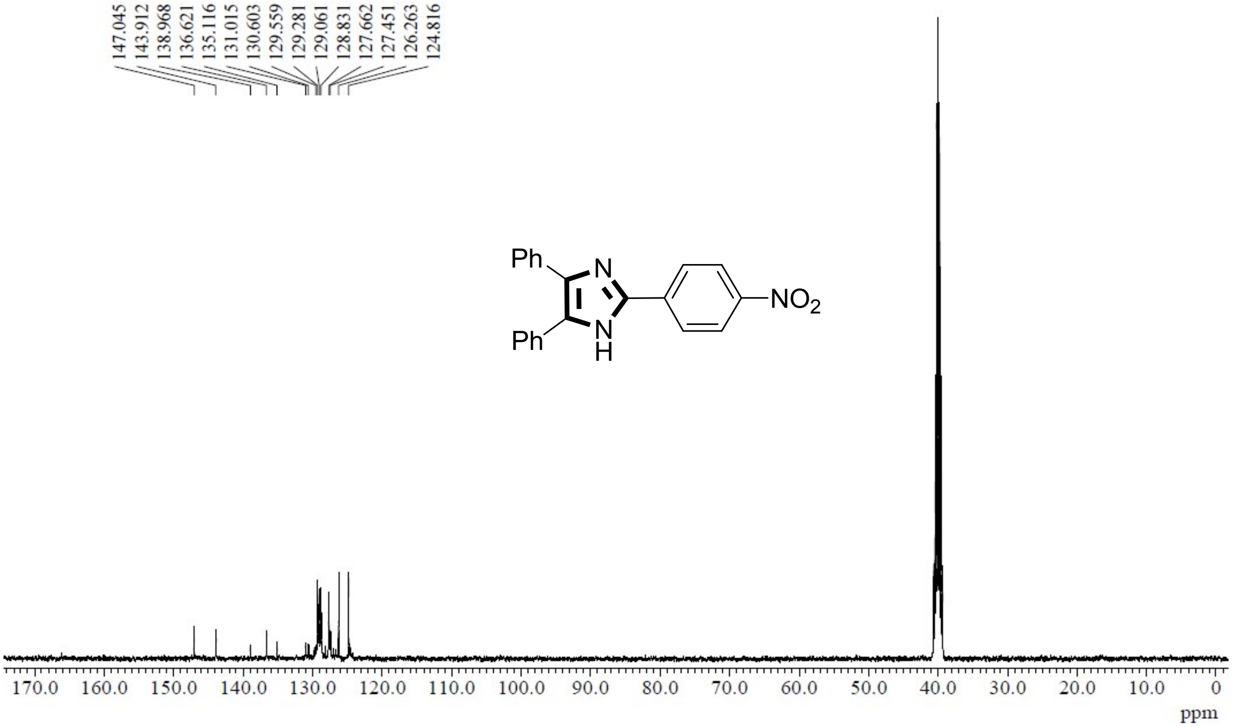

1.12 ^{1}H NMR of compound: **4g**

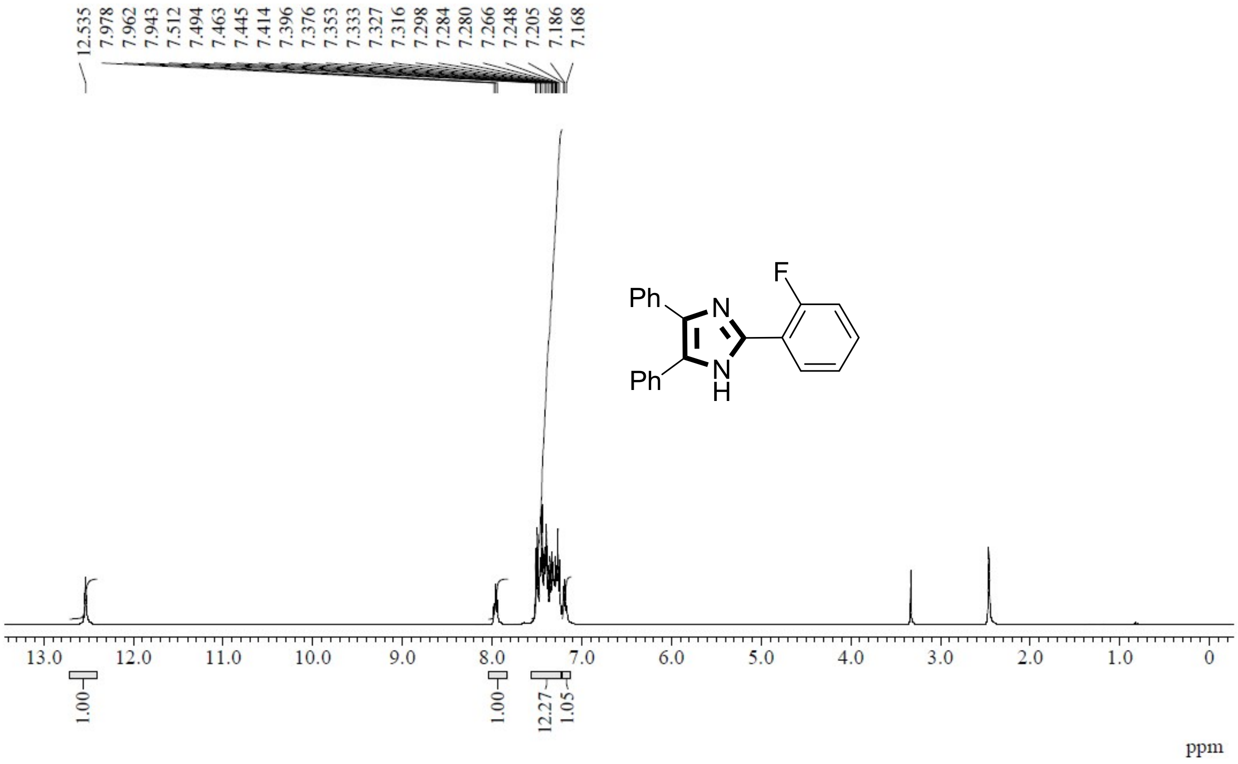

1.13 ^{13}C NMR of compound: **4g**

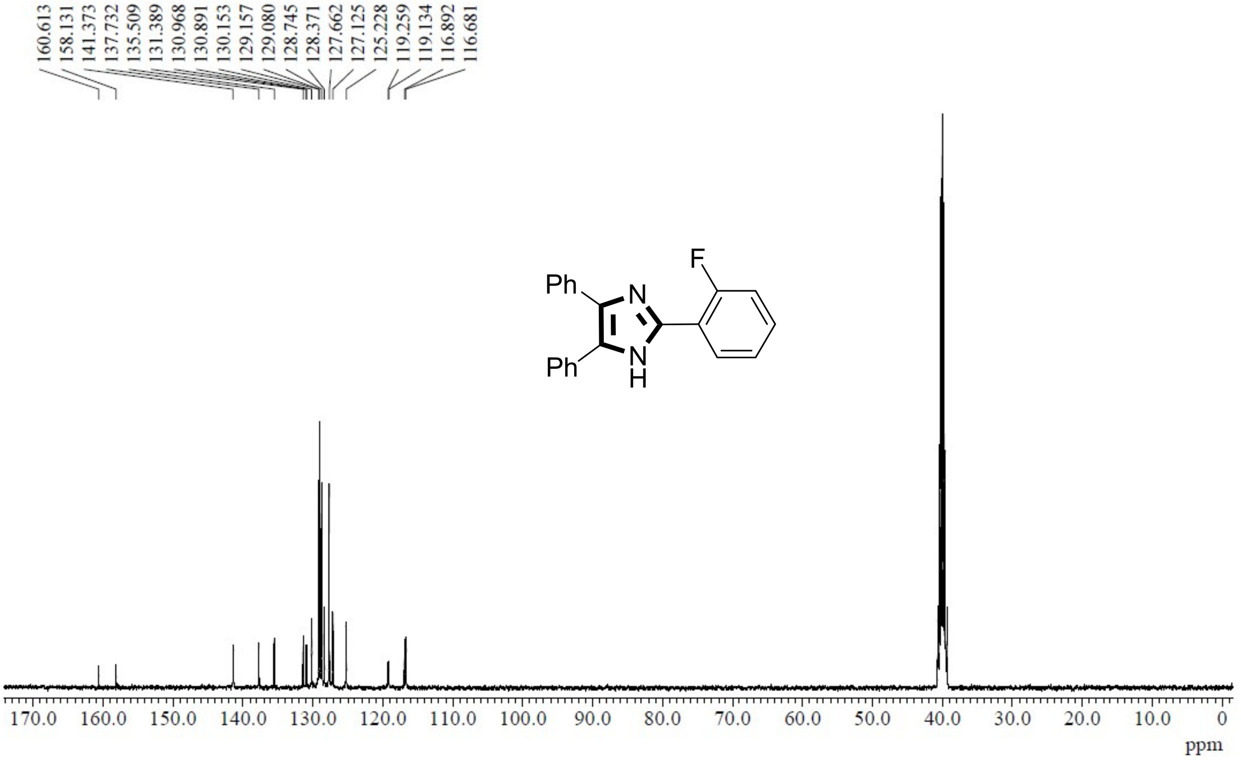

1.14 ^{1}H NMR of compound: **4h**

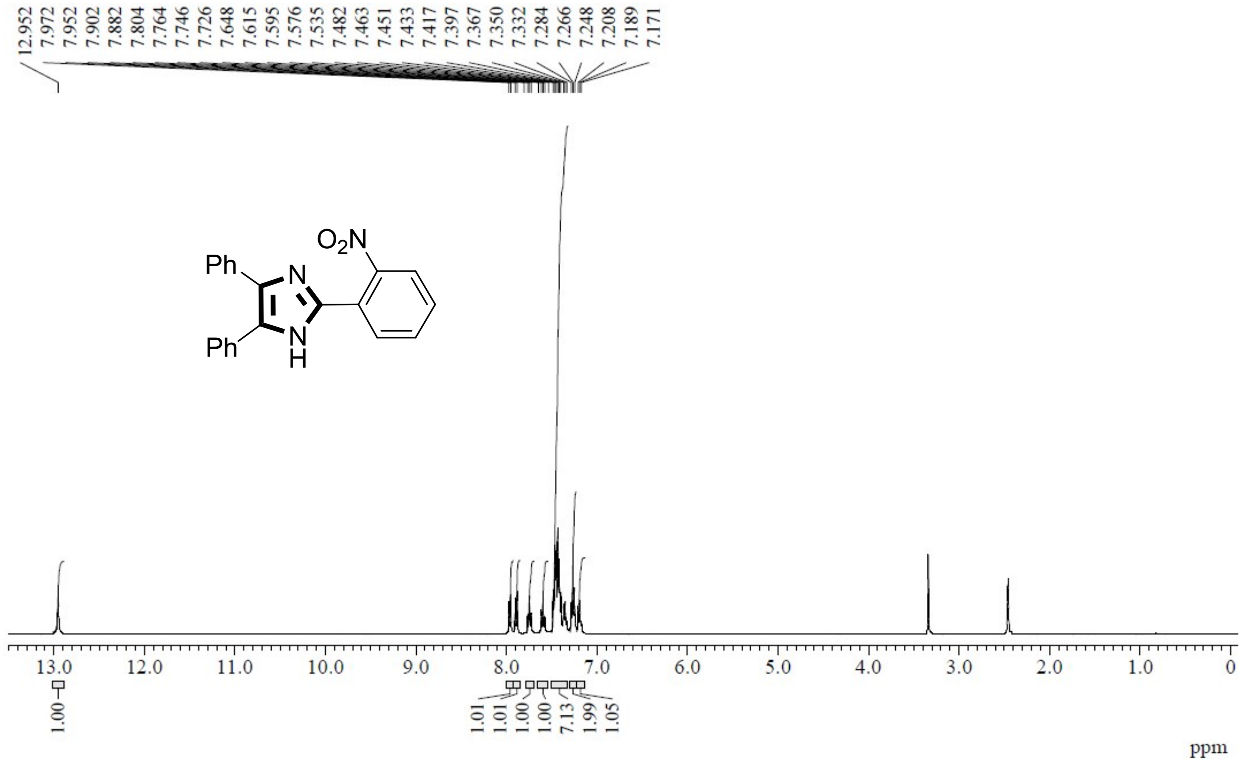

1.15 ^{13}C NMR of compound: **4h**

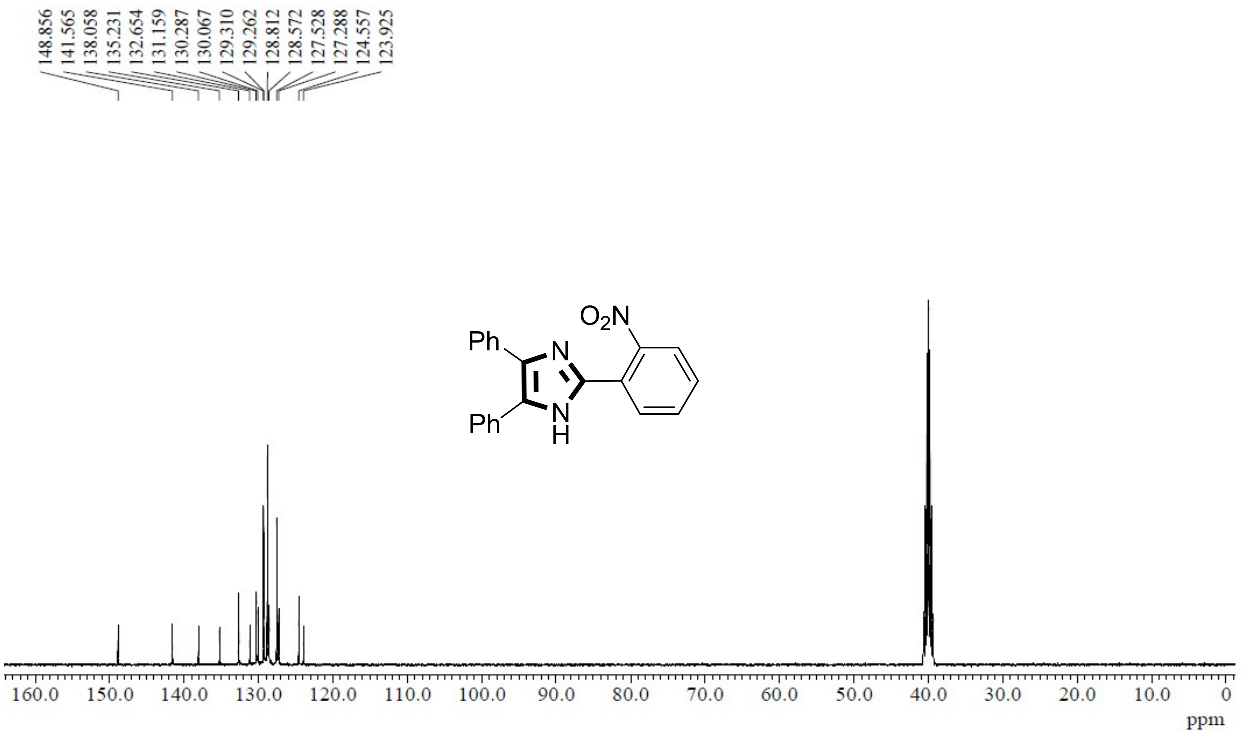

1.16 ^{1}H NMR of compound: **4i**

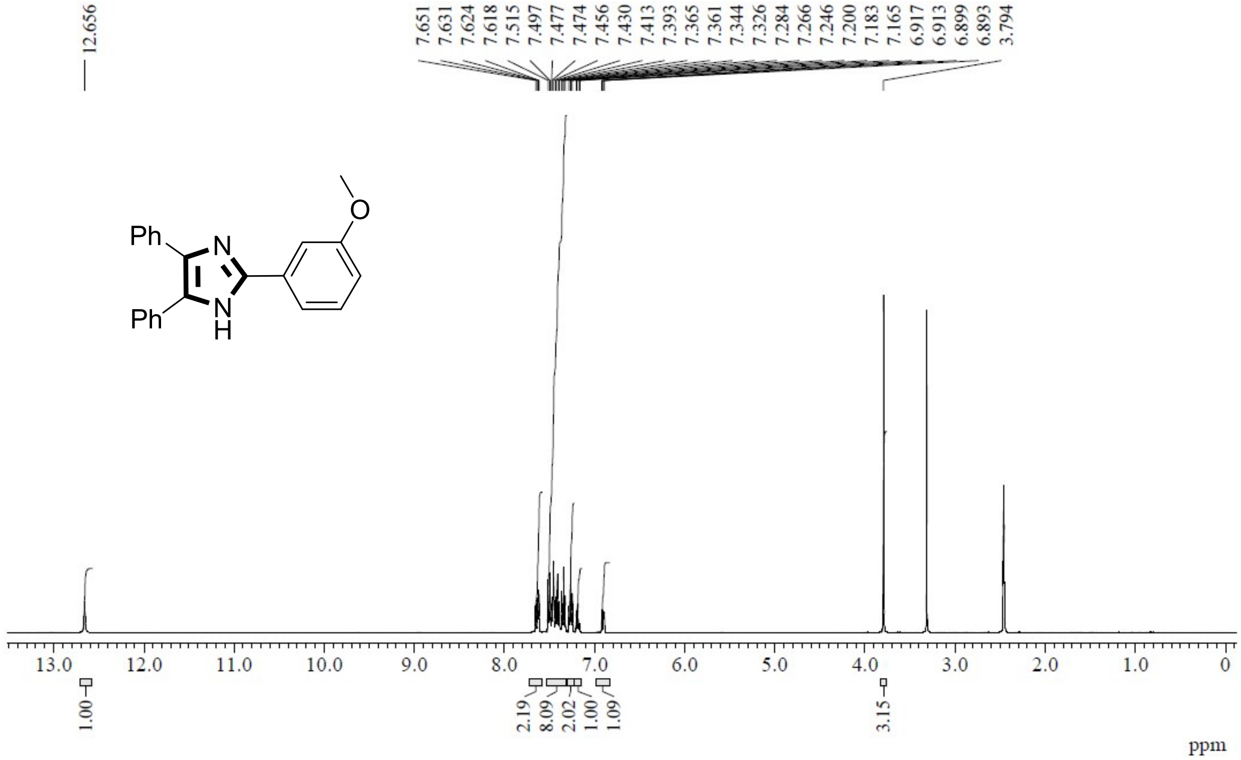

1.17 ^{13}C NMR of compound: **4i**

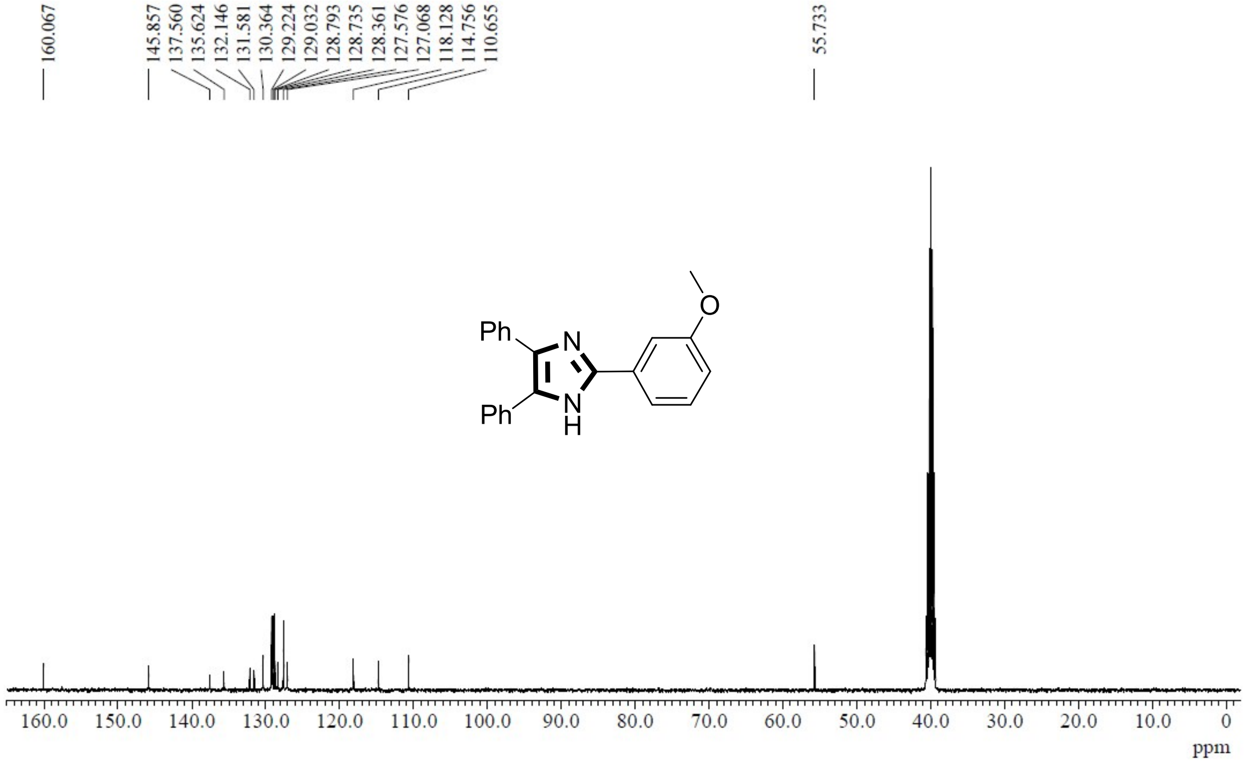

1.18 ^{1}H NMR of compound: **4j**

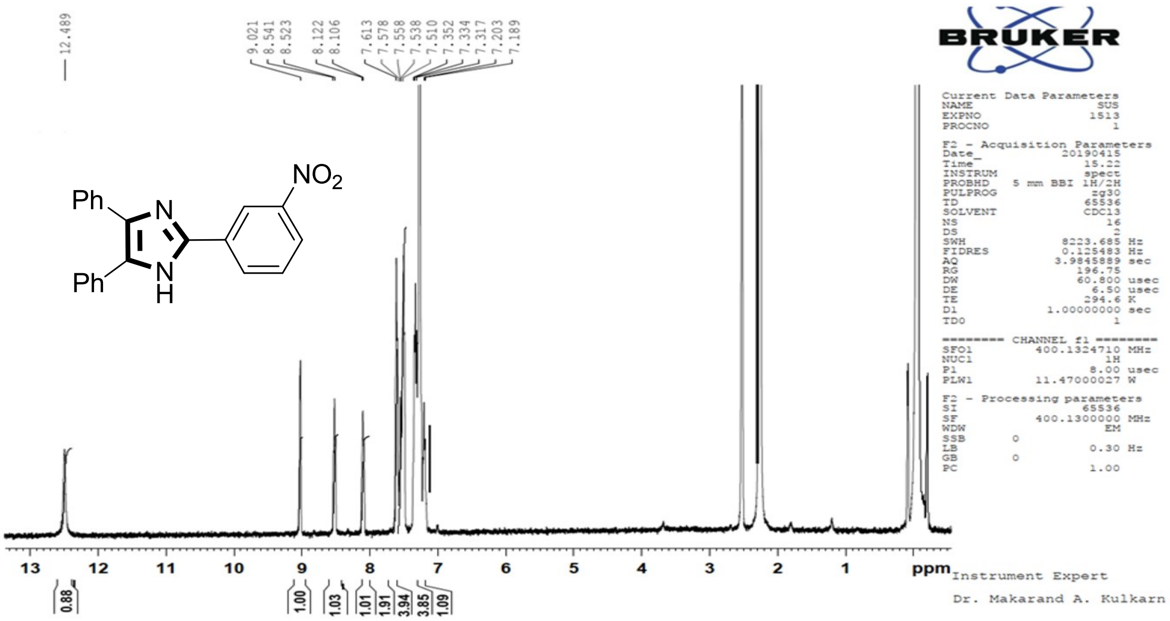

1.19 ^{1}H NMR of compound: **4k**

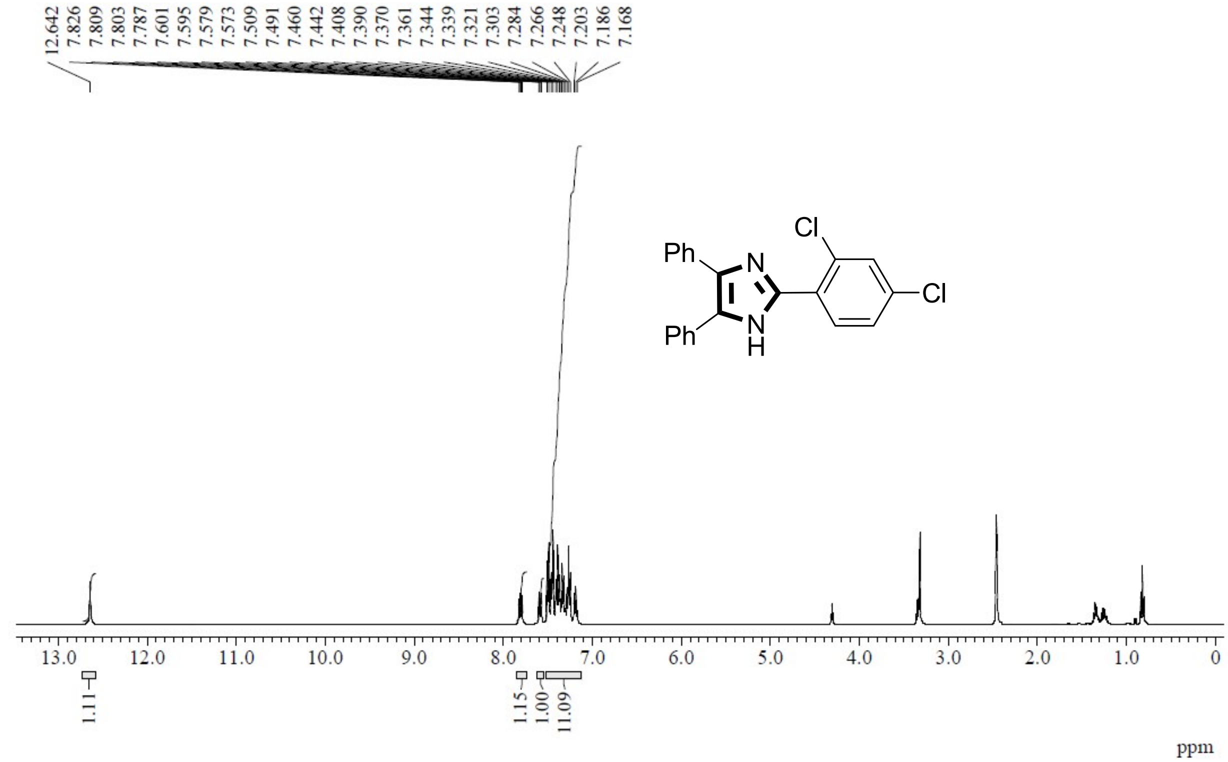

1.20 ^{13}C NMR of compound: **4k**

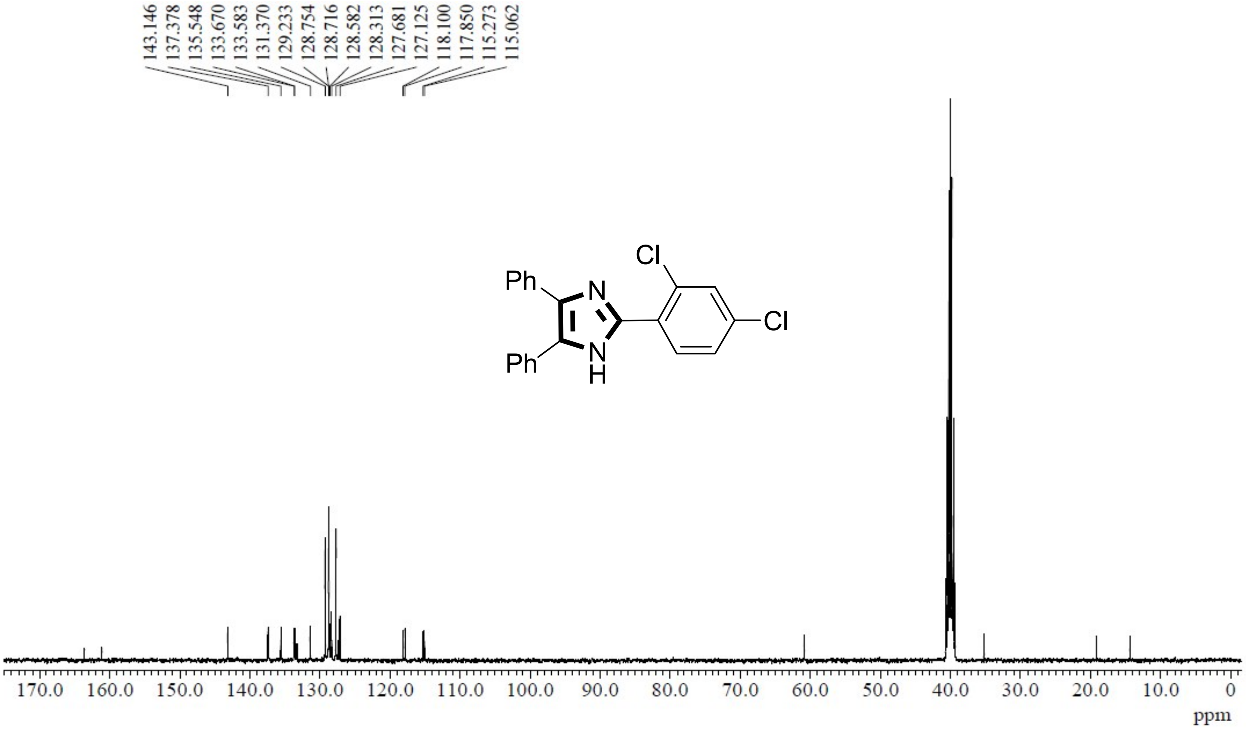

1.21 ^{1}H NMR of compound: **4l**

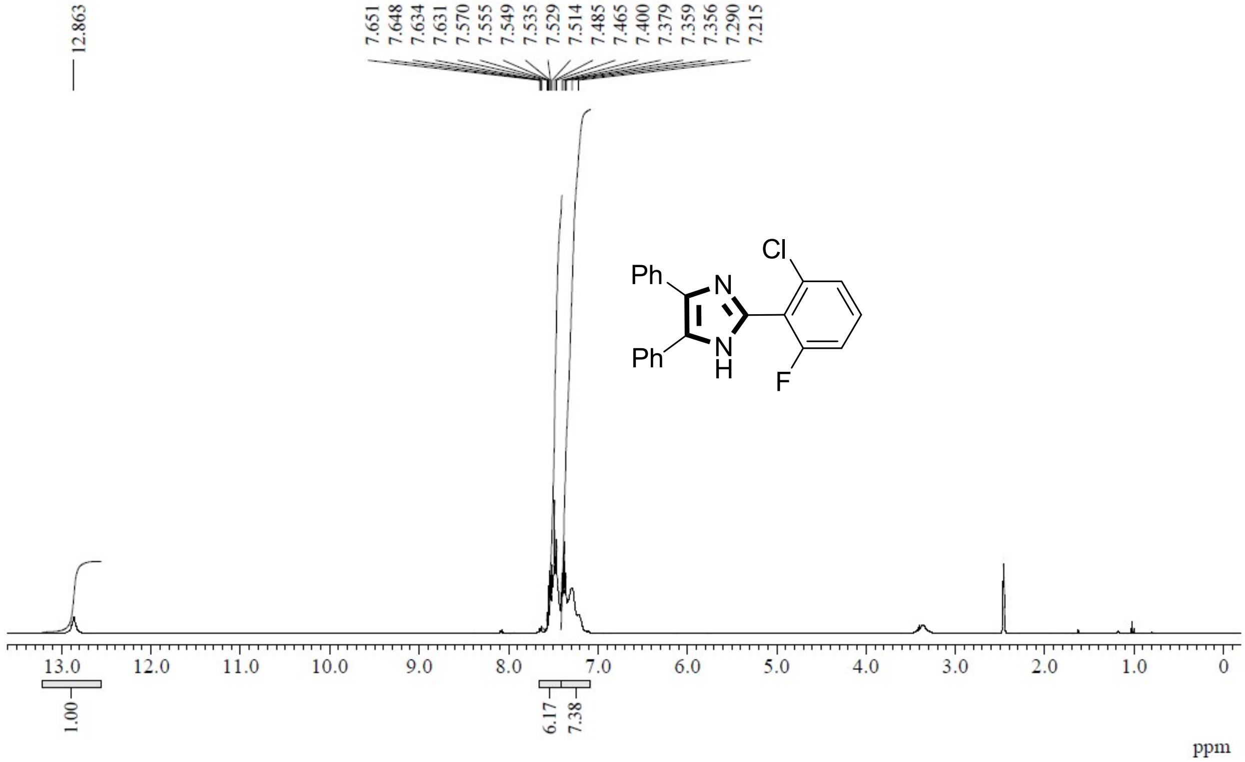

1.22 ^{13}C NMR of compound: **4l**

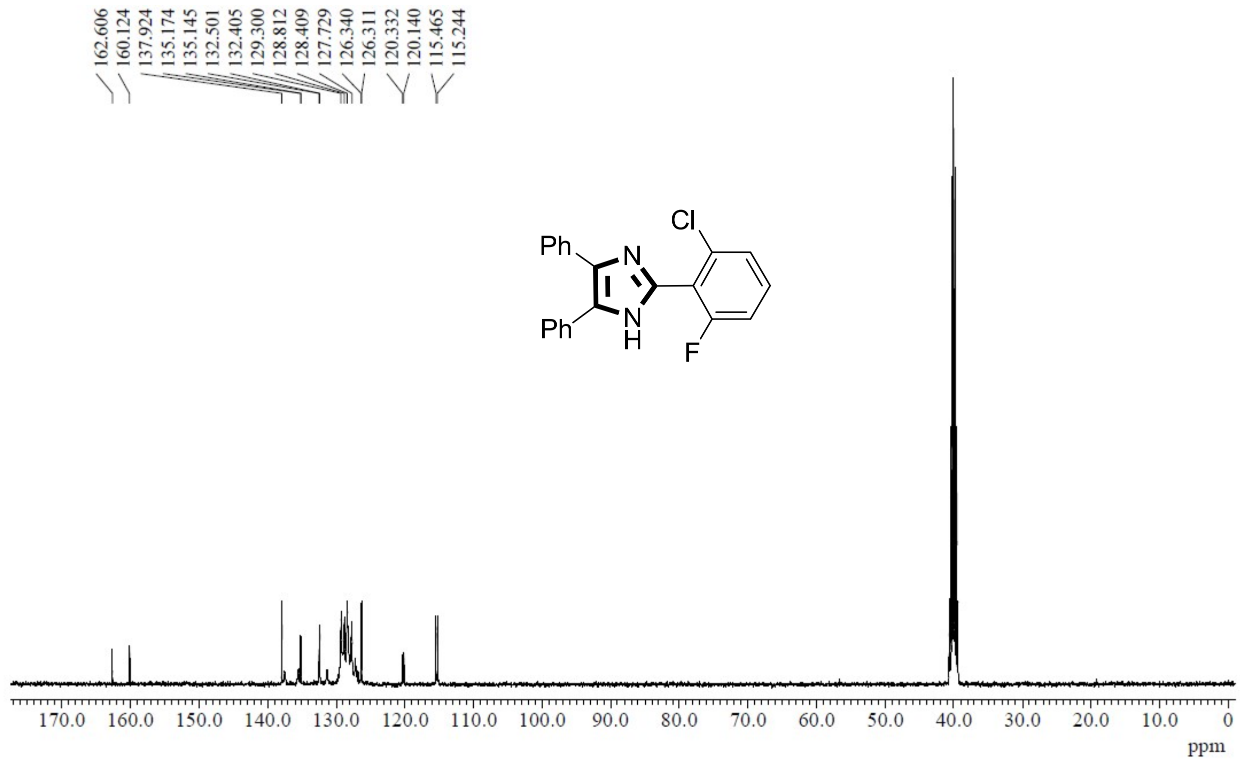

1.23 ^{1}H NMR of compound: **4m**

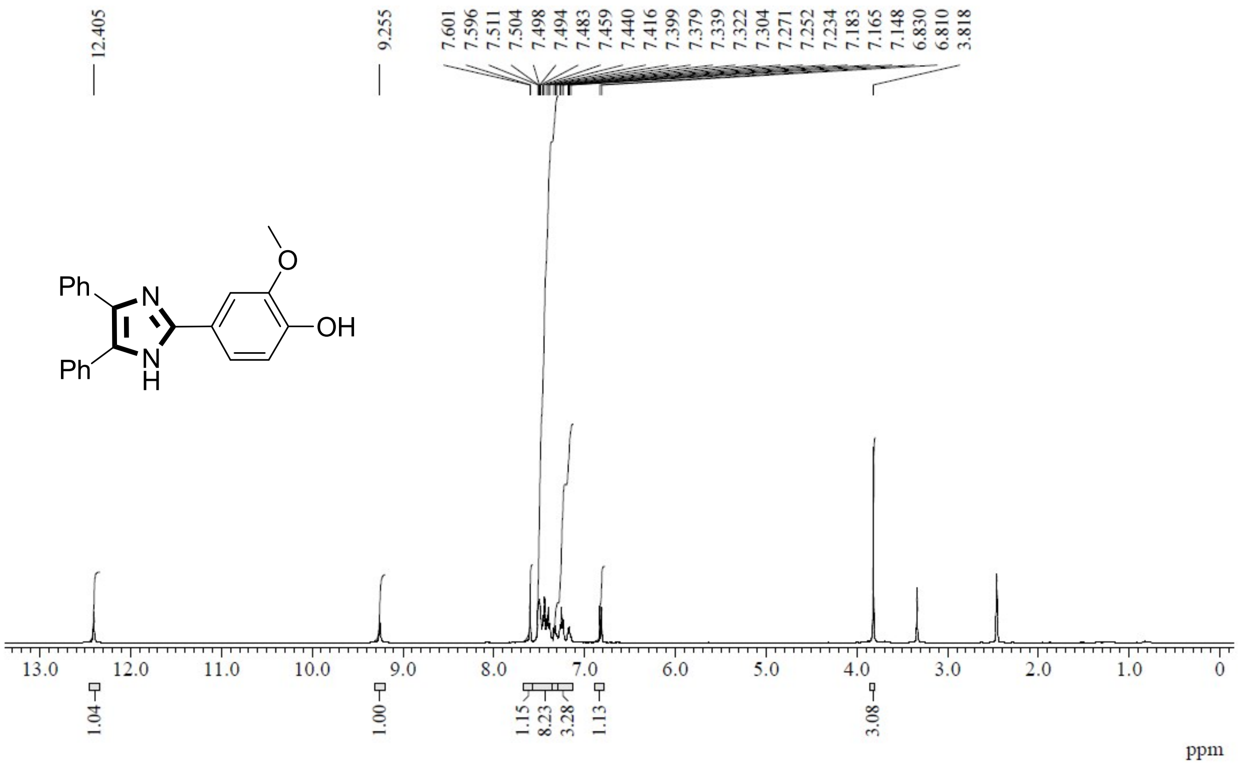

1.24 ^{13}C NMR of compound: **4m**

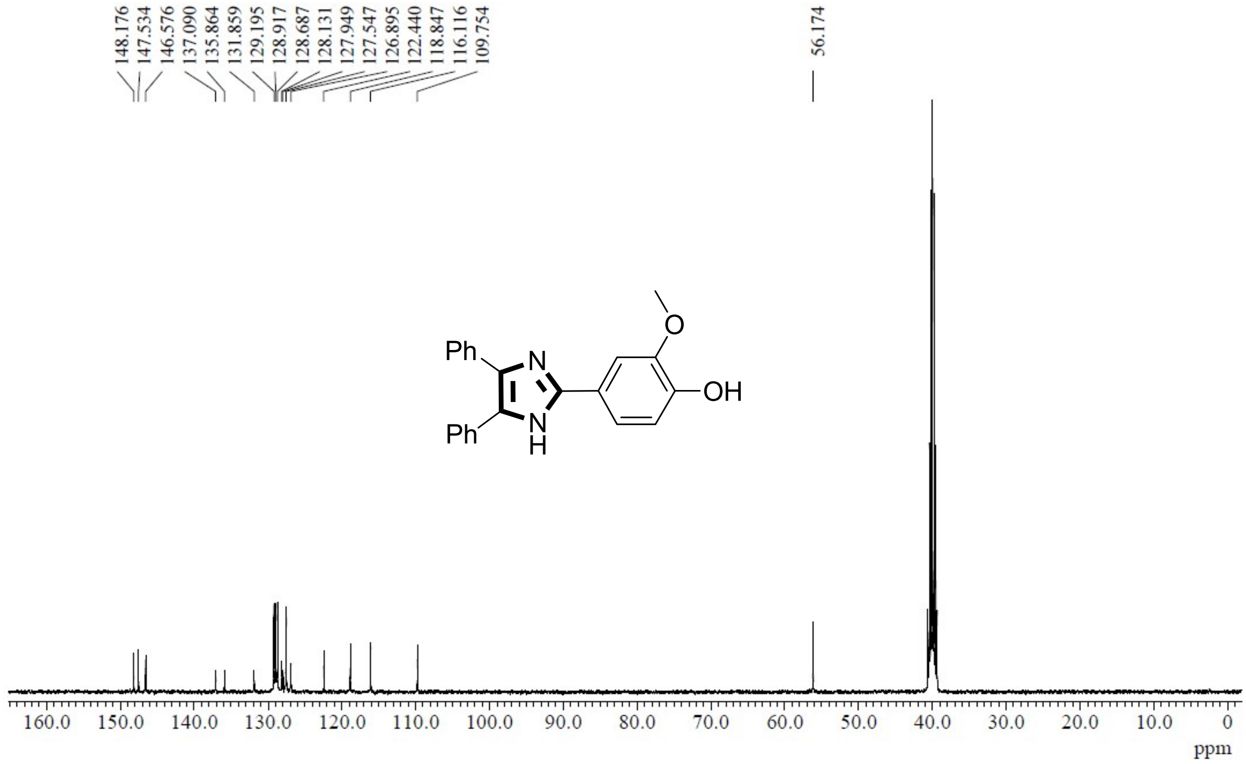

1.25 ^{1}H NMR of compound: **4n**

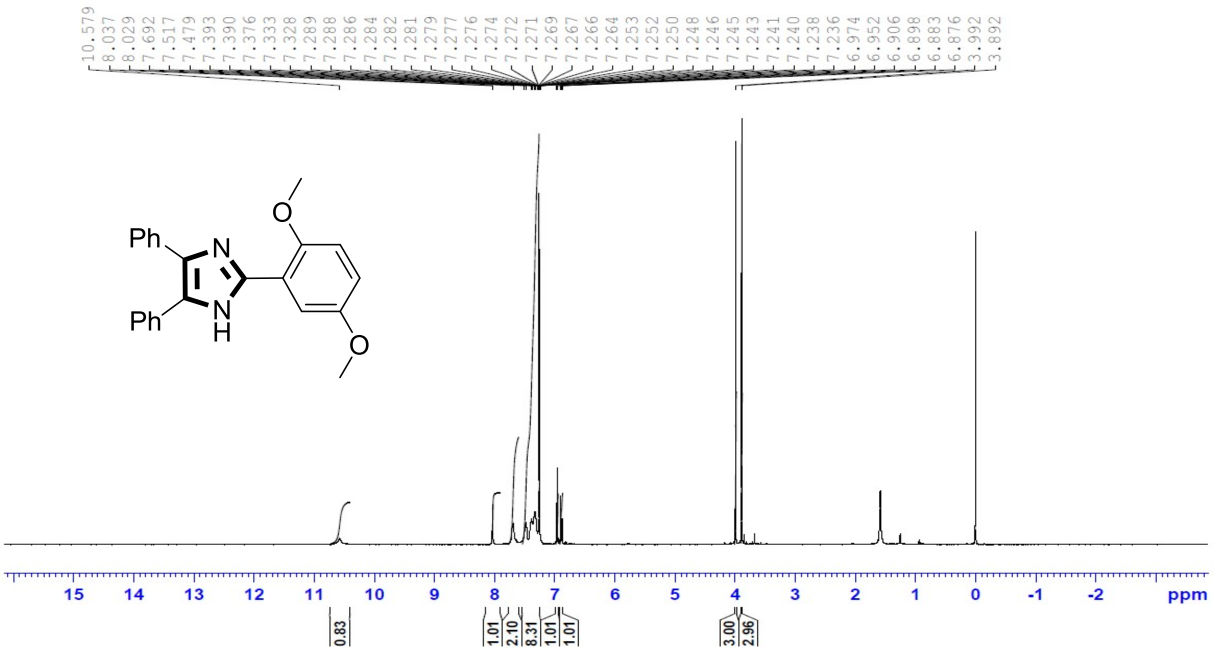

1.26 ^{13}C NMR of compound: **4n**

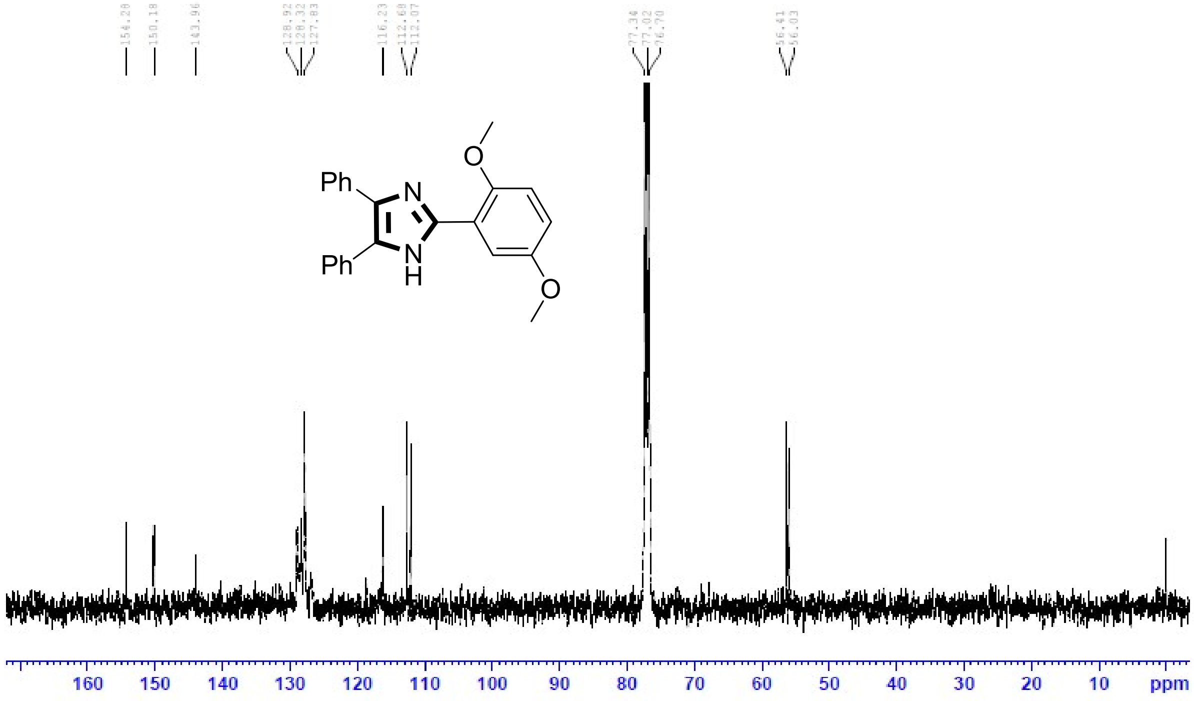

1.27 ^{1}H NMR of compound: **4o**

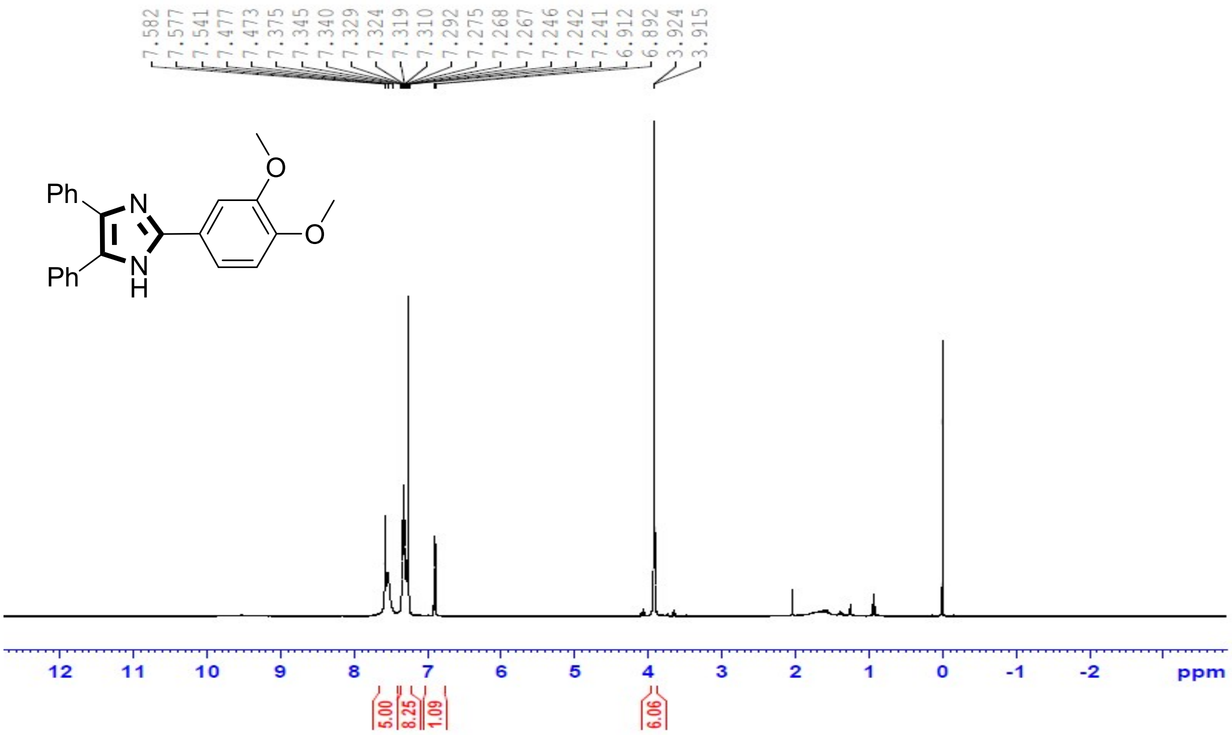

1.28 ^{13}C NMR of compound: **4o**

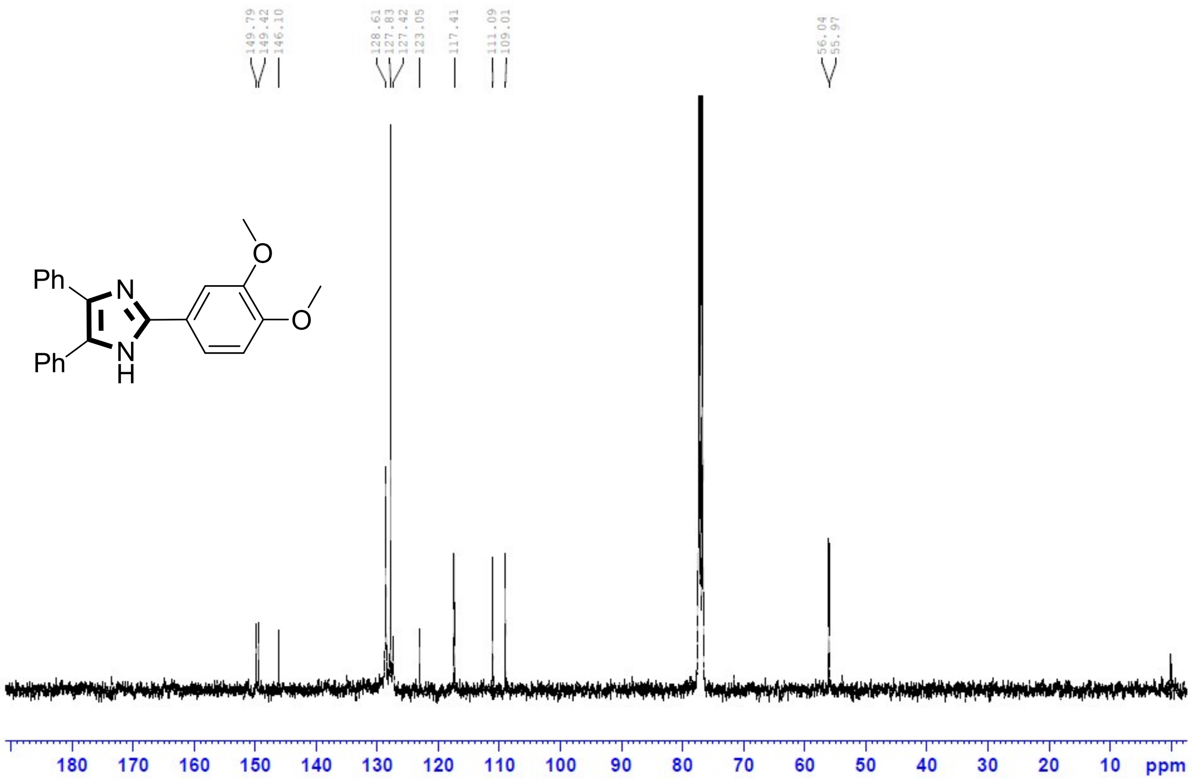

1.29 ^{1}H NMR of compound: **4p**

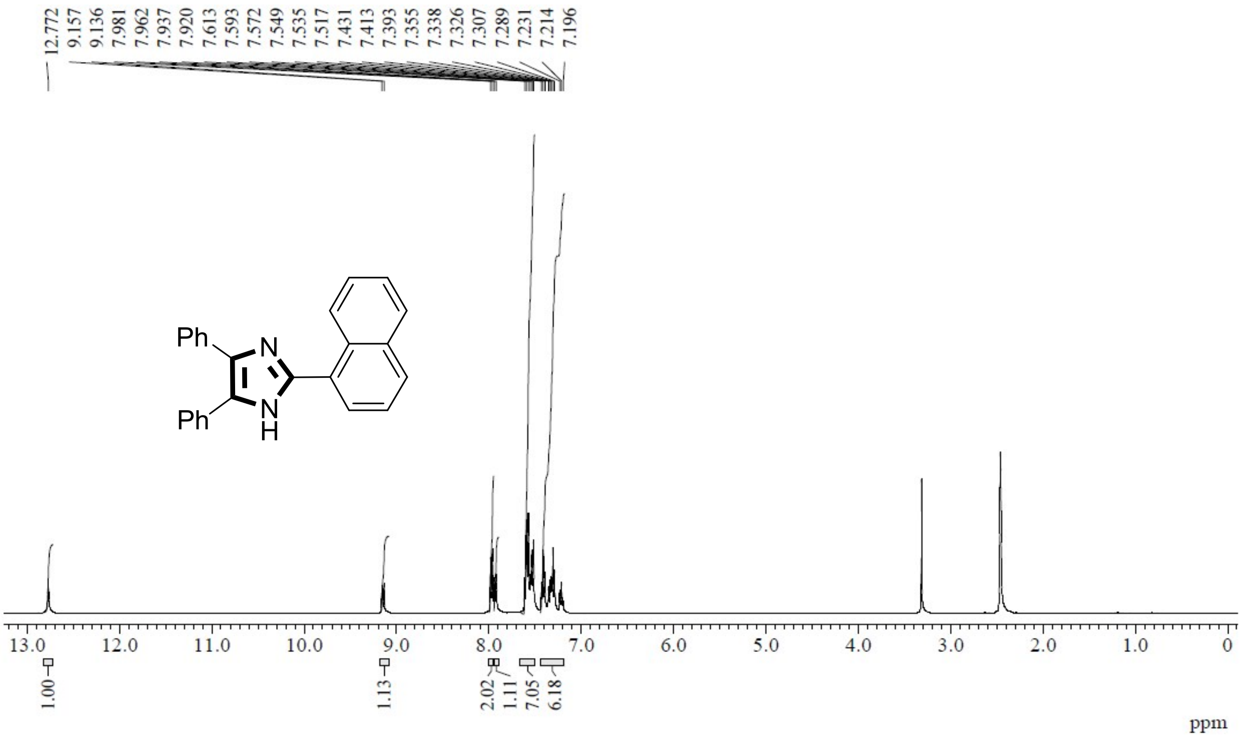

1.30 ^{13}C NMR of compound: **4p**

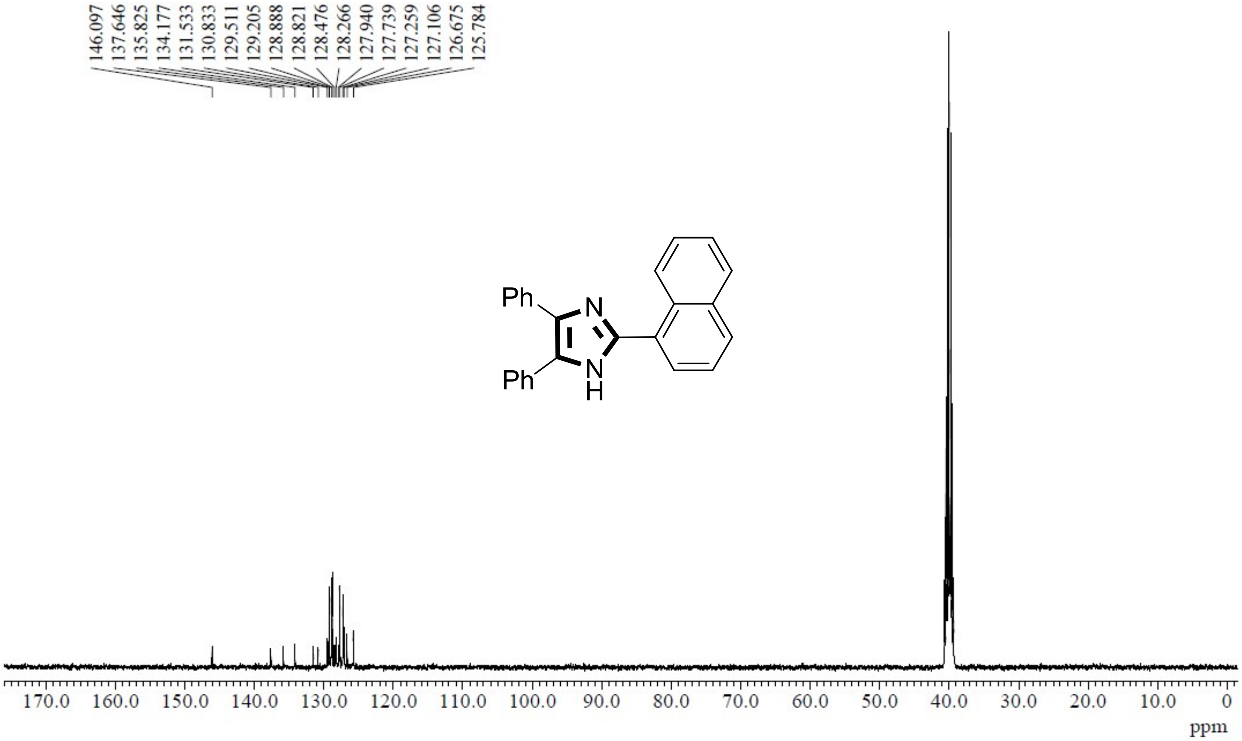

1.31 ^{1}H NMR of compound: **4q**

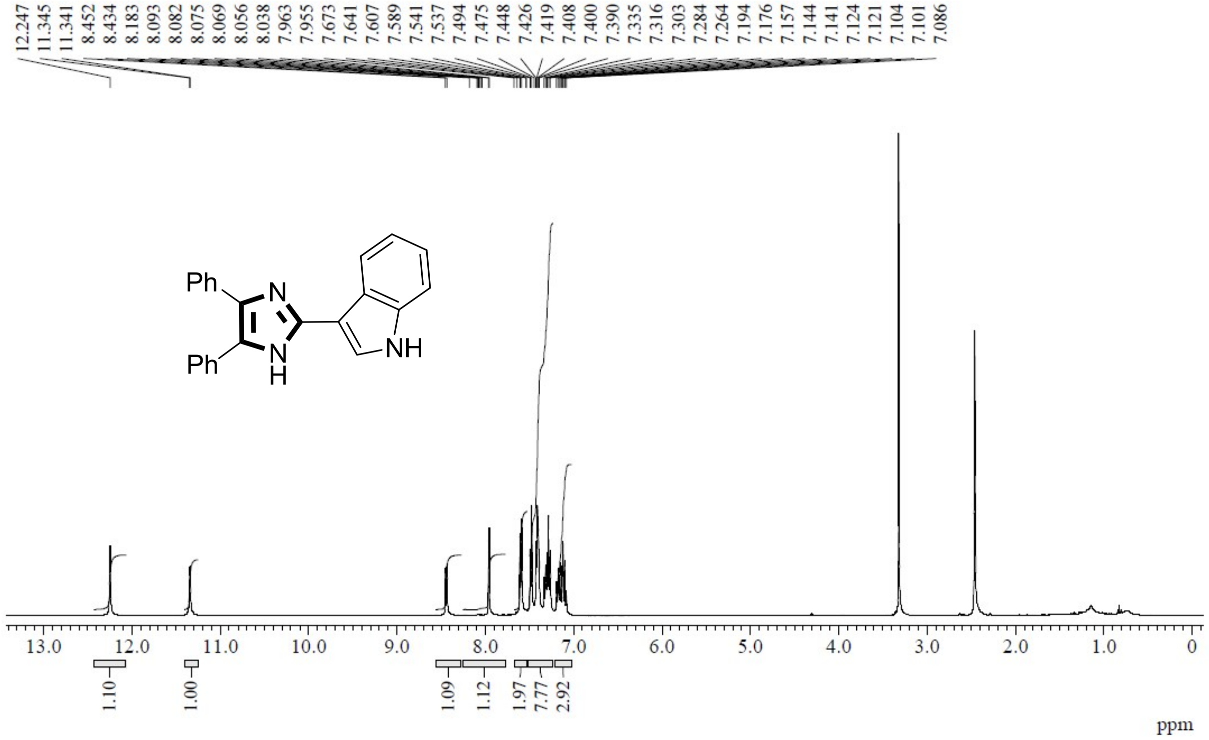

1.32 ^{13}C NMR of compound: **4q**

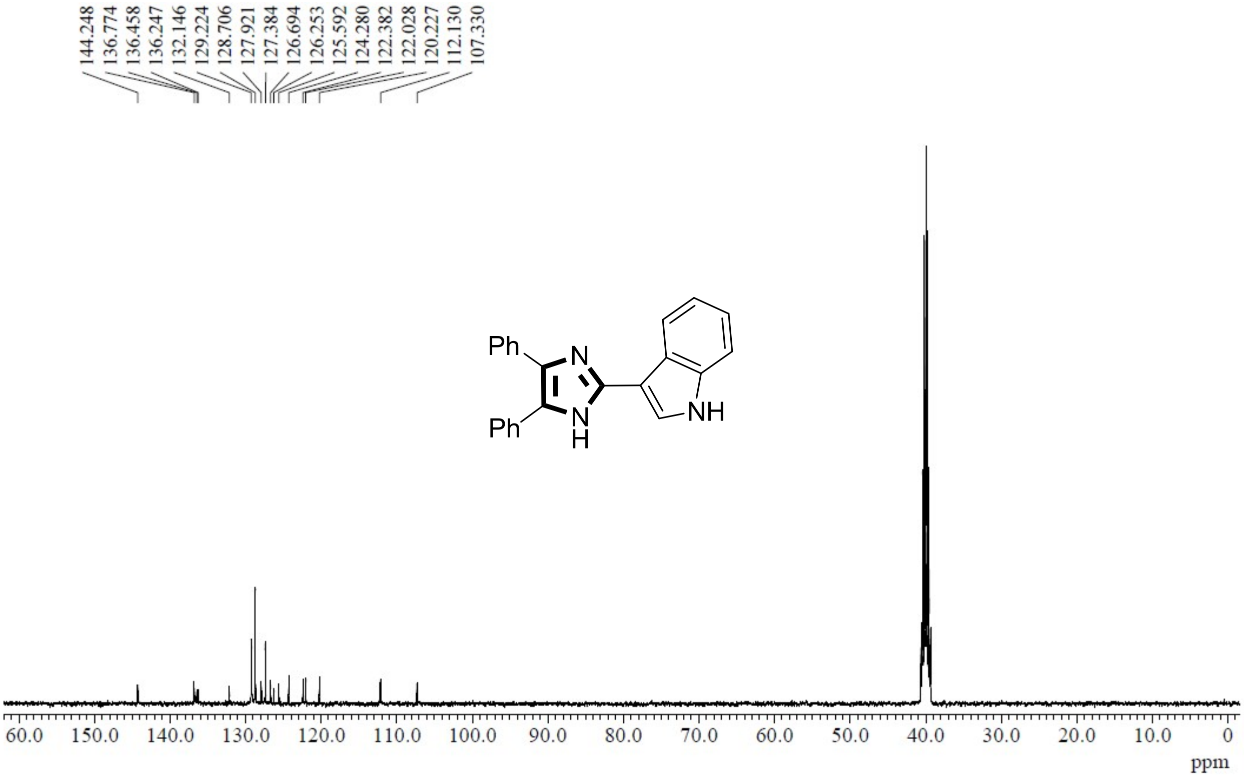

1.33 ^{1}H NMR of compound: **4r**

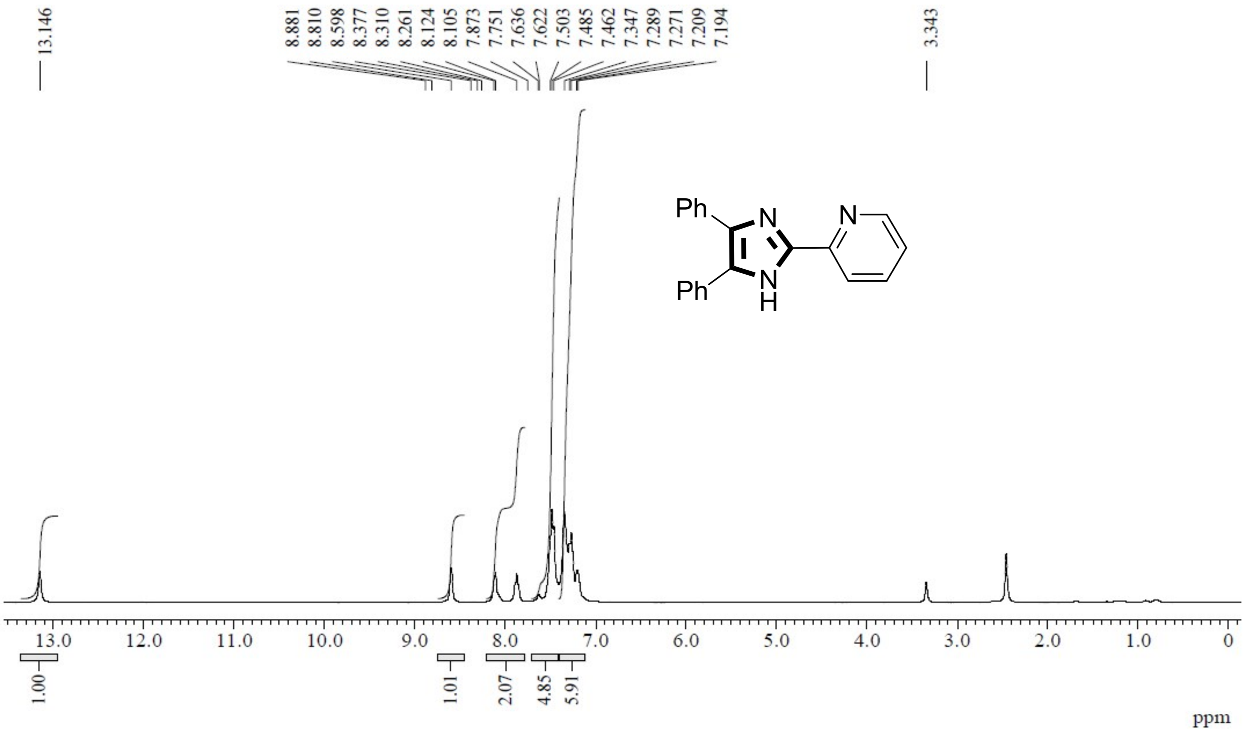

1.34 ^{13}C NMR of compound: **4r**

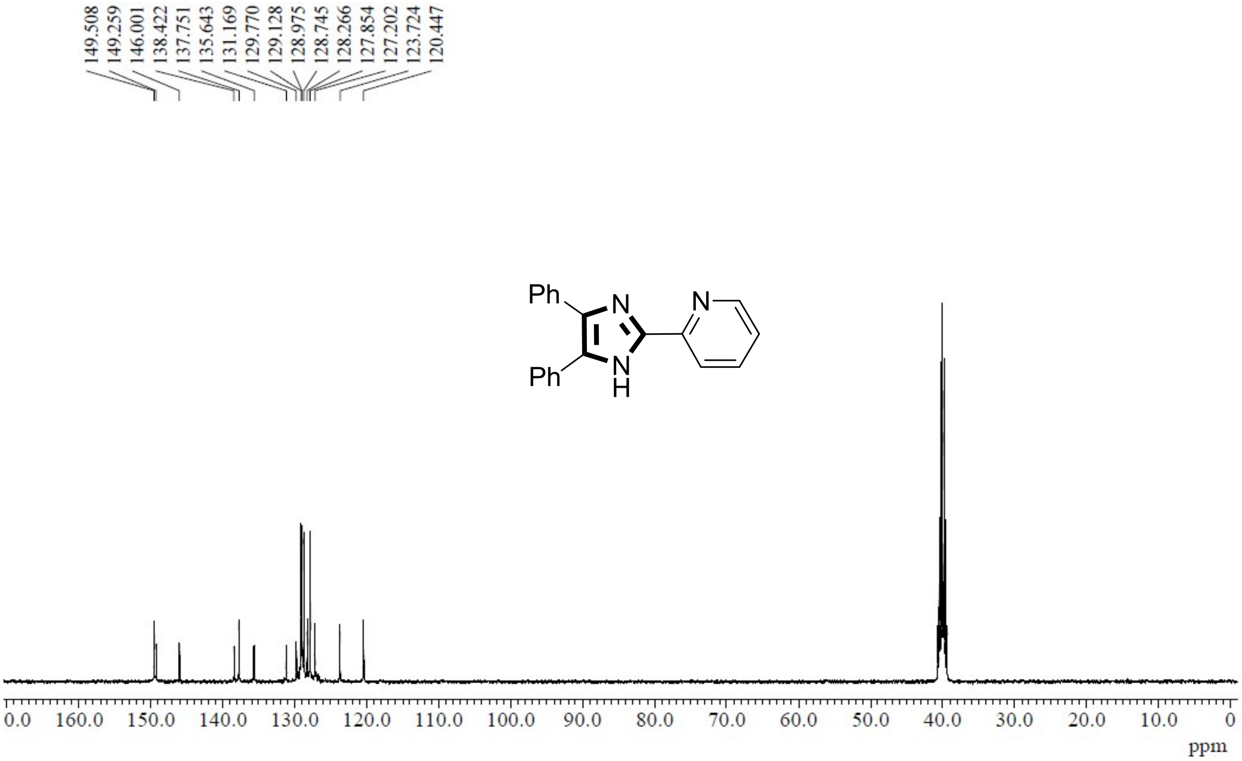

1.35 ^{1}H NMR of compound: **4s**

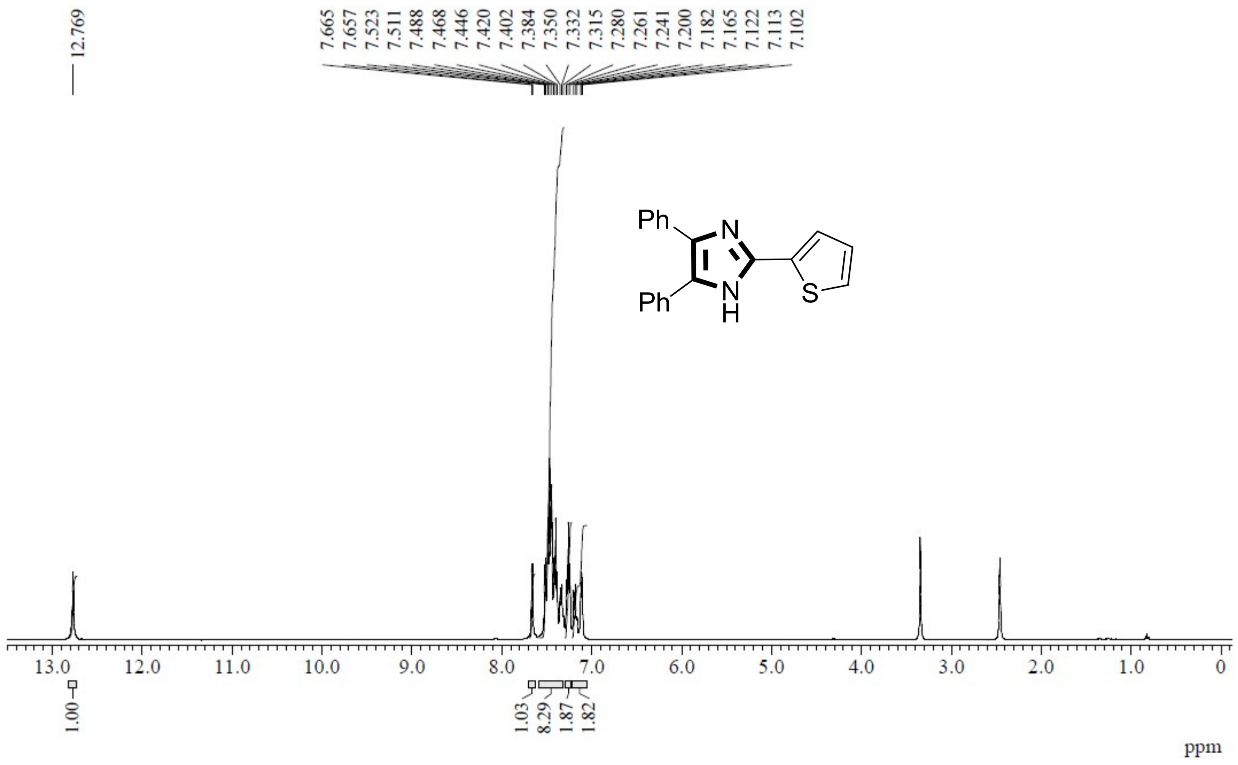

1.36 ^{13}C NMR of compound: **4s**

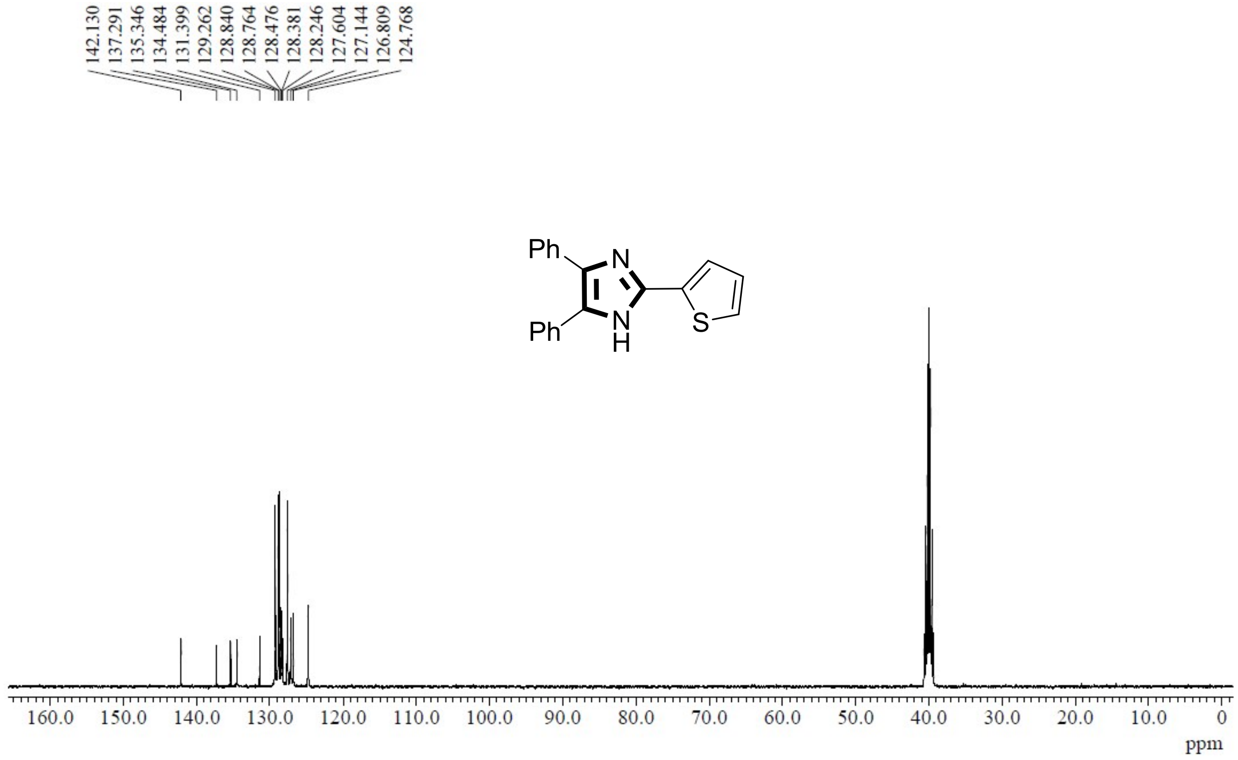

1.37 ^{1}H NMR of compound: **4t**

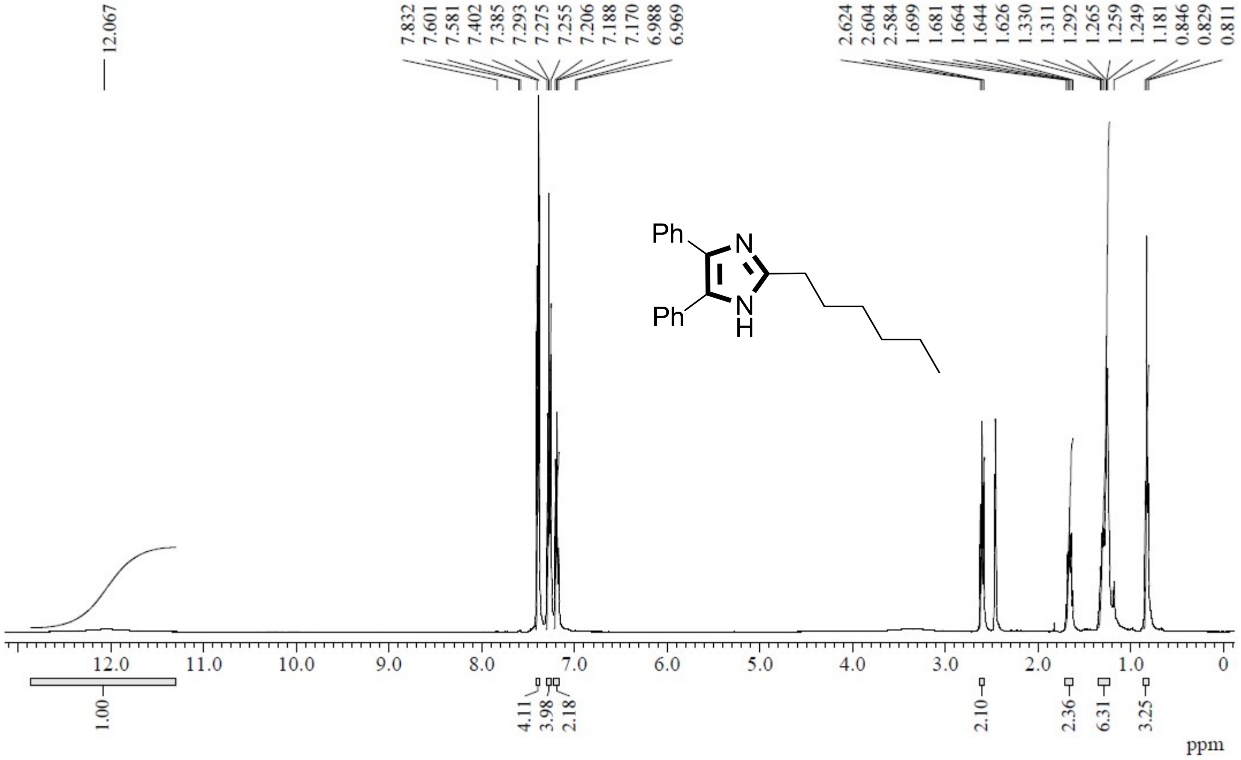

1.38 ^{13}C NMR of compound: **4t**

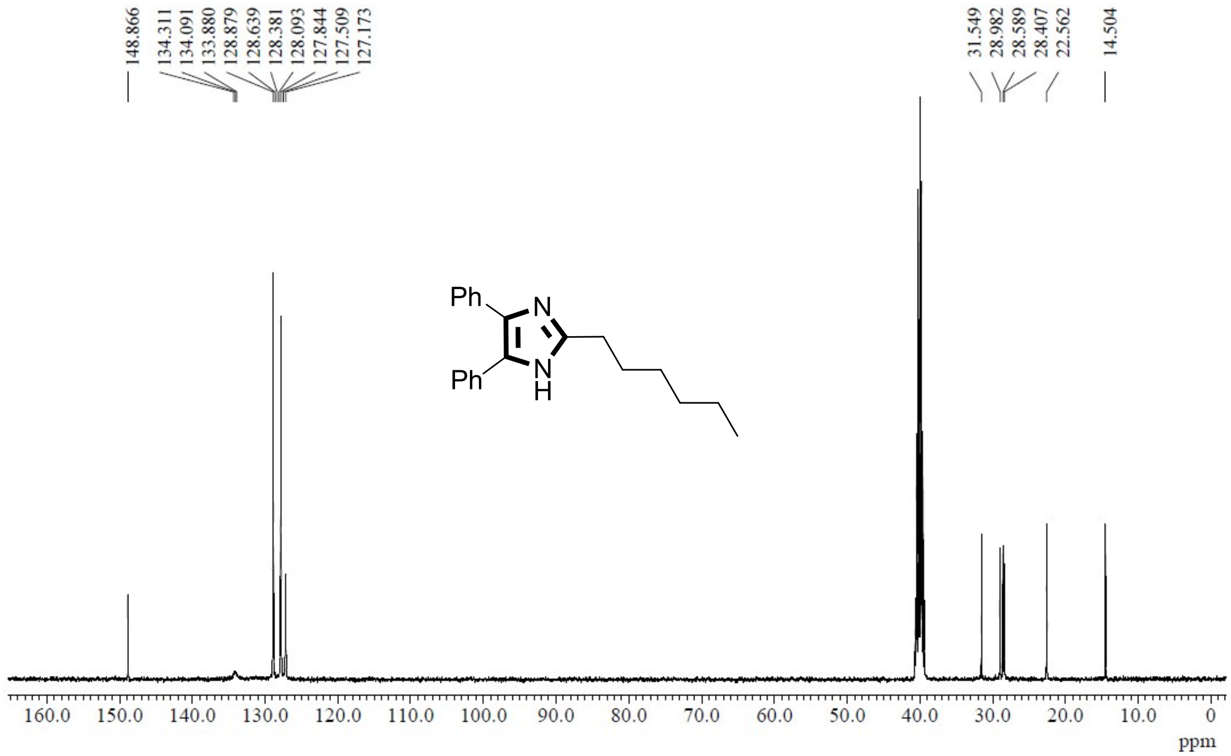

Supplement: RA-011-D1RA01767E-s001 [file RA-011-D1RA01767E-s001.pdf]
